# Reported community-level indoor residual spray coverage from two-stage cluster surveys in sub-Saharan Africa

**DOI:** 10.1186/s12936-017-1893-x

**Published:** 2017-06-13

**Authors:** David A. Larsen, Lauren Borrill, Ryan Patel, Lauren Fregosi

**Affiliations:** Department of Public Health, Food Studies and Nutrition, Syracuse University, Syracuse, NY USA

**Keywords:** Indoor residual spray, Community-level coverage, Implementation, Vector control

## Abstract

**Background:**

Malaria is an important cause of morbidity and mortality in malaria-endemic areas. Indoor residual spray is an effective intervention to control malaria, but high community-level coverage is needed to maximize its impact.

**Methods and results:**

Using thirty-four two-stage cluster surveys (e.g., demographic and health surveys) and lot quality assurance sampling, indoor residual spray was estimated at the community level (i.e. enumeration-area) across sub-Saharan Africa since 2010. For communities receiving indoor residual spray a logistic regression predicted whether community-level coverage exceeded 50% or not. Household-level coverage was equitable both in terms of wealth and urban/rural, with poorer and rural houses more likely to be sprayed than richer and urban houses. Coverage of indoor residual spray at the community level is poor across the continent, with 54% of communities receiving the intervention not reaching 50% coverage. Having >50% coverage at the community-level was not associated with increasing the number of houses sprayed in the country.

**Conclusions:**

Implementation and monitoring of indoor residual coverage at small geographical scales need to improve greatly to receive maximum benefit of the intervention.

**Electronic supplementary material:**

The online version of this article (doi:10.1186/s12936-017-1893-x) contains supplementary material, which is available to authorized users.

## Background

Malaria is a disease transmitted by mosquitoes of the *Anopheles* genus. While malaria is found throughout tropical regions of the world, sub-Saharan Africa carries the greatest malaria burden, with 90% of the estimated 212 million worldwide cases of malaria occurring in Africa in 2015 [1]. In the year 2000, malaria killed an estimated 1 million children [2]. Since 2000, malaria transmission in sub-Saharan Africa has been greatly reduced [3], primarily through the scale-up of insecticide-treated mosquito nets (ITN) [4]. Today ITNs are typically the primary intervention to control malaria vectors in malaria-endemic countries, while indoor residual spray (IRS) is most often used as a supplemental vector control if used at all.

Beginning in 1955, the Global Malaria Eradication Campaign used IRS with the insecticide DDT as the primary vector control method, but the environmental risks associated with DDT and growing insecticide resistance caused the need to move away from using DDT for IRS [5]. The implementation of IRS regained popularity with the establishment of the President’s Malaria Initiative (PMI) in 2005 [6]. Today protecting an individual from malaria transmission with IRS costs three times more than protecting an individual from malaria transmission with ITNs [7]. IRS may take more prominence in the future of malaria control due to increasing pyrethroid resistance [8, 9]. Furthermore, malaria control programmes may consider combining IRS and ITNs to increase impact beyond that obtained by a single intervention. Currently it remains unclear whether IRS in the context of high ITN coverage decreases malaria transmission further than high coverage of either intervention alone [10–12].

IRS typically kills malaria vectors after they have taken a blood meal. Although IRS is deployed within households, a high community-level coverage is required to ensure effective intervention deployment. The World Health Organization (WHO) has suggested a threshold of 85% of houses being covered by IRS to achieve a successful campaign [13], however the science of the dose–response relationship between community-level coverage of IRS and protective efficacy is woefully inadequate. A single study conducted on Bioko Island found that houses sprayed with IRS saw no benefit of the intervention with community-coverage below 20%, and houses not sprayed with IRS saw benefit of IRS if the community was covered at 80% or above [14]. Unfortunately, none of the recent randomized controlled trials of IRS report on spatial IRS coverage. Furthermore the “coverage” indicator currently accepted by the largest IRS programmes is not a community-level coverage at all but rather a household-level acceptance rate of IRS (Bridges et al. under review).

This paper uses two-stage cluster surveys to estimate community-level coverage of IRS in sub-Saharan Africa since 2010 and find factors that may be associated with effective community-level coverage.

## Methods

### Data sources/measurement

All two-stage cluster surveys were considered for the analysis that were (1) performed in sub-Saharan Africa in 2010 or later were, (2) measured indoor residual spray coverage, and (3) publicly available in October 2016. Specifically, Demographic and Health Surveys (DHS), Malaria Indicator Surveys (MIS), AIDS Indicator Surveys (AIS) and Multiple Indicator Cluster Surveys (MICS) were examined for suitability. These surveys are typically powered to determine child mortality and fertility trends at the regional or provincial level. The samples are selected in two stages. First standard enumeration areas typically defined by the country’s Central Office of Statistics and based upon the previous census are selected probability proportionate to size. The size of enumeration areas varies, but would typically not be larger than a few square kilometres. Second, households are randomly selected within each selected enumeration area. For the remainder of this manuscript the enumeration areas will be referred to as community.

### Potential bias

Questions exist about the ability of nationally-representative surveys to measure IRS coverage with accuracy. In particular, the wording of the survey has been called into question as respondents may confuse “sprayed the walls in the house with insecticide” with a self-application of commercially-bought sprays such as Doom or Raid (Kilian, pers. comm). This type of misclassification error is present with all analyses of survey data, and is likely to be randomly distributed throughout the survey. To account for the potential misclassification bias of including community in the analysis that was not targeted for IRS intervention, the analysis of EA-level IRS coverage was limited to EAs where at least 5 households reported receiving IRS. Furthermore, the EA-level analysis of community-level coverage of IRS was limited to houses reporting they were sprayed by a government or non-governmental organization.

### Quantitative variables

The surveys ask two separate questions about IRS coverage, first whether the house was sprayed in the previous 12 months and second who conducted the spraying (government, NGO, or private). To determine the drivers of household-level coverage of IRS households were categorized as covered by IRS if they reported being sprayed in the previous 12 months without regard to who conducted the sprayed. To determine the drivers of community-level coverage of IRS households were categorized as covered by IRS if they reported being sprayed in the previous 12 months and reported that the spraying was done by either the government or an NGO.

In addition to the spray variables numerous potential factors associated with IRS coverage were considered a priori, namely: wealth quintile, urban or rural, the number of children in the household under 5 years of age, and the number of women in the household of reproductive age (15–49 years old). These factors were aggregated to the community as mean wealth quintile, urban or rural, mean number of children under 5 years of age per household, and mean number of women of reproductive age per household.

### Lot quality assurance sampling—community-level IRS coverage

Although the surveys are not powered to estimate IRS coverage within the primary sampling unit, lot quality assurance sampling (LQAS) can give a probability of a community surveyed achieving a threshold coverage such as the 85% threshold recommended by the WHO [13]. LQAS is commonly used to estimate vaccination coverage [15], a similar intervention to IRS in that population-level coverage is perhaps more important for preventing transmission of disease than individual or household-level coverage [16]. For IRS, the malaria vector is killed while resting on the wall of a household after the mosquito has taken a blood meal thereby making household-level coverage somewhat beneficial to neighbours and community-level coverage more important for controlling malaria transmission.

Three separate thresholds were set for IRS success at the community level: at least 50% coverage, at least 75% coverage and at least 85% coverage as recommended by the WHO [13]. Equation 1 presents the probability that a given community with a number of sampled houses (n) would achieve a threshold (p) given the number of sampled houses reporting their house being sprayed the previous month (a). Probabilities of misclassification <10% were deemed acceptable, and EAs were categorized as having no sprayed houses, <50% coverage, 50–75% coverage, 75–85% coverage, and >85% coverage.

1 $$P(a) = \frac{n!}{a!(n - a)!}p^{a} q^{n - a}$$

where p = the proportion of houses sprayed in the community in previous 12 months, q = (1−p) or the proportion of houses not sprayed in the community in the previous 12 months, n = the number of houses samples in the EA, a = the number of houses in the sample sprayed in the previous 12 months, and n−a = the number of houses in the sample not sprayed in the previous 12 months.

The ability to predict >85% coverage is partially dependent upon the number of households sampled within an EA. Predicting >85% coverage with 90% confidence requires a minimum of 15 houses sampled within an EA, and only at 23 houses sampled within a community can a single house not report IRS coverage and the community still be classified at >75% coverage (for a spreadsheet on LQAS decisions see Additional file 1). Therefore, the analysis was limited to EAs where information on IRS was known for at least 15 houses.

### Statistical methods—household-level analysis

To assess household-level drivers of IRS coverage first an equity analysis at the community-level was conducted wherein household-IRS coverage was estimated as a function of both wealth quintile and urban/rural for each survey identified. The pooled data were then use to regress the probability of a house receiving IRS as a function of household wealth quintile, household ownership of at least one ITN, urban or rural, number of children under the age of five (categorized as 0, 1, or 2+), and the number of women of reproductive age (categorized as 0, 1, or 2+). A logistic regression was used with the community as a random intercept and included dataset as a covariate.

### Statistical methods—community-level analysis

To assess community-level IRS coverage across sub-Saharan Africa EAs were first described as having no IRS coverage (no houses reporting IRS performed by the government or an NGO), <50% IRS coverage (at least 5 houses reporting IRS performed by the government or an NGO but unable to exceed the 50% threshold as set by LQAS), 50–75% IRS coverage (a sufficient number of houses reporting IRS performed by the government or an NGO to meet the 50% threshold as set by LQAS), 75–85% coverage (a sufficient number of houses reporting IRS performed by the government or an NGO to meet the 75% threshold as set by LQAS), or >85% coverage (a sufficient number of houses reporting IRS performed by the government or an NGO to meet the 85% threshold as set by LQAS). EAs were then categorized as having <50% coverage or >50% coverage (excluding EAs reporting no IRS) and the probability of a community having >50% coverage was regressed on community-level factors of wealth, urban or rural, number of children under 5 years of age, and number of women of reproductive age, and ITN coverage. A logistic regression was used with robust standard errors adjusted for clustering at the level of the survey dataset and included household-level IRS coverage for the survey as a covariate categorized as <10%, 10–20%, 20–30%, and >30%. The number of households sampled in the community was also included as a covariate to determine the influence that sample size had on a community being classified as >50% coverage.

## Results

### Participants

**Fig. 1:**
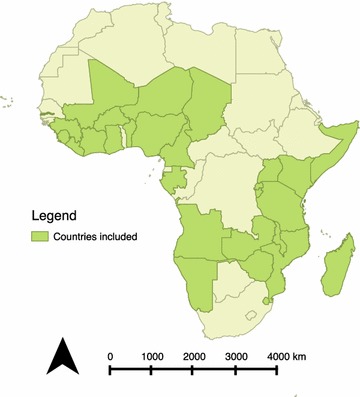
Countries with data included in the analysis

IRS was not measured in MICS. Of the remaining DHS, MIS and AIS 42 datasets publicly available as of December 2016 were identified, of which 34 datasets had a measurement of indoor residual spray. Nations represented in this analysis are displayed in Fig. 1.

### Descriptive data

**Fig. 2:**
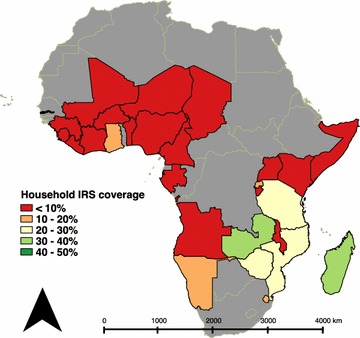
Proportion of households reporting IRS in the most recent survey

Measured household-level IRS coverage ranged from <1% of households in numerous surveys (Burkina Faso 2014 MIS, Cameroon 2011 DHS, Cote d’Ivoire 2011 DHS, Guinea 2012 DHS, Kenya 2014 DHS, Niger 2012 DHS, and Nigeria 2010 MIS) to 41% measured in the Gambia 2013 DHS (Fig. 2). Of 3641 EAs reporting any house receiving IRS performed by the government or NGOs, 1631 (45%) had fewer than 5 houses reporting coverage and were excluded from EA-level analysis.

### Household-level predictors of IRS

**Fig. 3:**
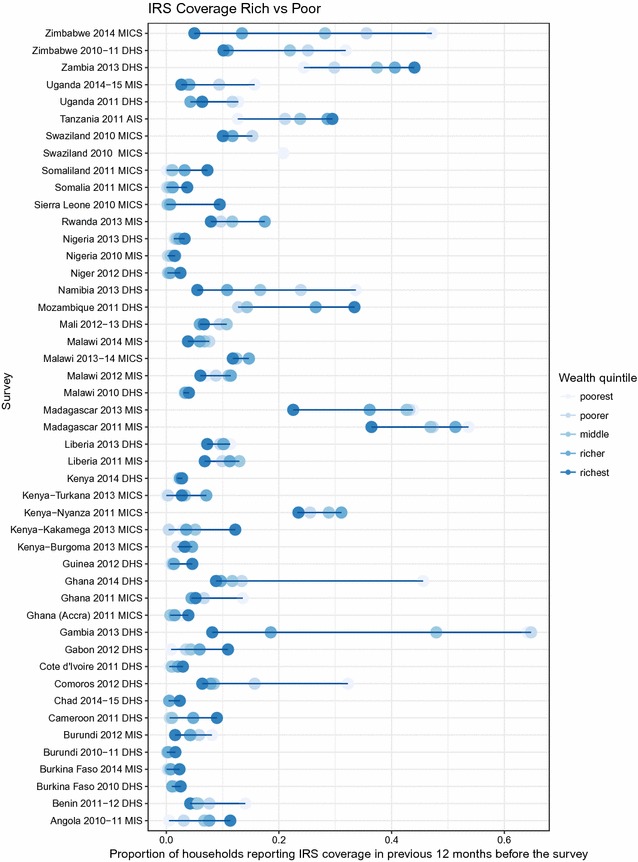
Equity of IRS coverage between rich and poor as measured by national household survey

**Fig. 4:**
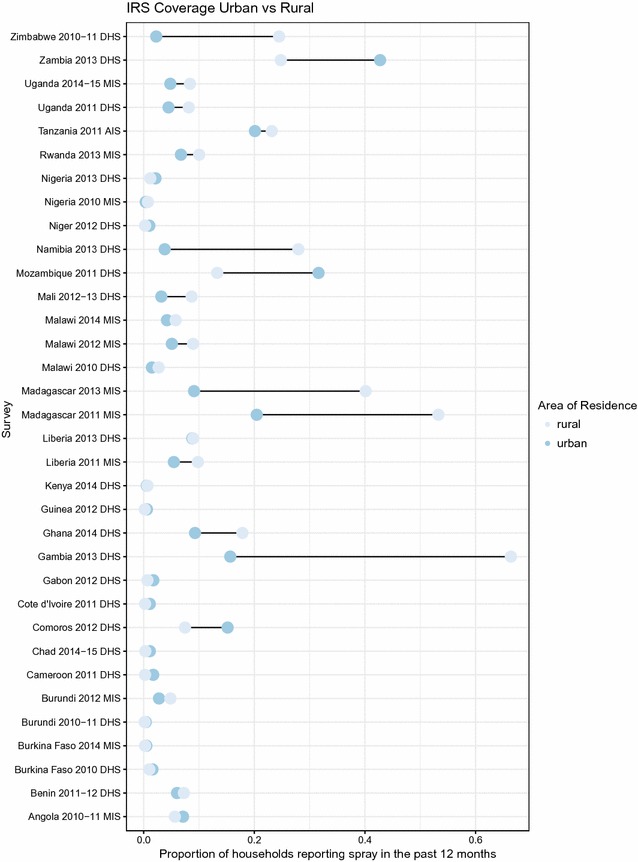
Equity of IRS coverage between urban and rural as measured by national household survey

Equity of IRS differed by country, with IRS coverage in various countries higher in the lower wealth quintiles than the higher wealth quintiles (Fig. 3). Of the 34 surveys with IRS coverage Y% reported higher coverage in the lowest wealth quintile compared to the highest quintile. Considering urban and rural equity, 59% of surveys reported higher coverage in rural areas and 41% of surveys (14) reported higher coverage in urban areas (Fig. 4).

Table 1 presents results on factors associated with households reporting IRS in the previous 12 months. In general, poorer and rural households were more likely to receive IRS, and IRS was more likely in households with more children under 5 years of age, more women of reproductive age and households that also report having an ITN.

**Table 1:** Factors associated with households reporting IRS in the previous 12 months

Factor	Categorization	OR (95% CI)	P value	AOR (95% CI)	P value
Wealth	Quintile 1	Reference	Reference	Reference	Reference
	Quintile 2	0.80 (0.75–0.85)	<0.0001	0.79 (0.74–0.85)	<0.0001
	Quintile 3	0.74 (0.68–0.81)	<0.0001	0.76 (0.70–0.83)	<0.0001
	Quintile 4	0.69 (0.63–0.76)	<0.0001	0.76 (0.69–0.84)	<0.0001
	Quintile 5	0.55 (0.50–0.61)	<0.0001	0.64 (0.56–0.73)	<0.0001
Location	Urban	Reference	Reference	Reference	Reference
	Rural	1.48 (1.34–1.63)	<0.0001	1.24 (1.09–1.39)	0.001
Children under 5	None	Reference	Reference	Reference	Reference
	One in household	1.23 (1.19–1.28)	<0.0001	1.16 (1.12–1.20)	<0.0001
	Two or more in household	1.46 (1.40–1.53)	<0.0001	1.28 (1.22–1.33)	<0.0001
Women of reproductive age	None	Reference	Reference	Reference	Reference
	One in household	1.13 (1.09–1.17)	<0.0001	0.98 (0.94–1.03)	0.436
	Two or more in household	1.34 (1.28–1.41)	<0.0001	1.22 (1.16–1.29)	<0.0001
ITN ownership	None	Reference	Reference	Reference	Reference
	One or more in household	1.42 (1.34–1.52)	<0.0001	1.39 (1.30–1.49)	<0.0001

Both unadjusted and adjusted models included dataset as a covariate and standard errors were adjusted for correlated data at the community level

n = 360,089 households, 14,939 EAs

### Community-level predictors of high IRS coverage

**Fig. 5:**
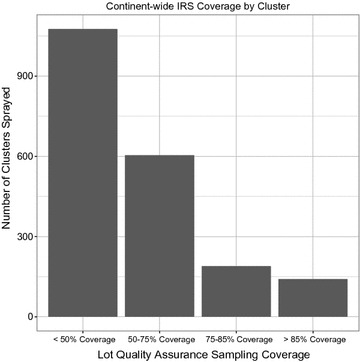
Estimated IRS coverage within EAs where at least 5 households in the community reported IRS in the previous 12 months. See additional files for individual-survey results

Among 2010 EAs where at least 5 houses reported being sprayed in the previous 12 months 54% (1076) missed the 50% LQAS threshold and 16% (330) were able to be classified as >75% coverage (Fig. 5). EAs located in rural areas and with higher numbers of children were more likely to achieve a 50% threshold (Table 2). Achieving higher coverage at the national level was not necessarily associated with achieving higher spatial coverage at the EA-level. EAs in countries with 20–30% household IRS coverage were more likely to achieve >50% coverage than EAs in countries with <10% household IRS coverage (adjusted odds ratio [AOR] = 4.7, 95% confidence interval [CI] = 3.1–7.1), however EAs in countries with 10–20% or >30% were no more likely to achieve >50% than EAs in countries with household IRS coverage <10%. Individual country-level maps are available in Additional files 2–28.

**Table 2:** Factors associated with EAs achieving at least 50% coverage in the previous 12 months

Factor	Categorization	OR (95% CI)	P value	AOR (95% CI)	P value
Wealth	Continuous	0.64 (0.49–0.84)	0.002	0.77 (0.55–1.08)	0.125
Location	Urban	Reference	Reference	Reference	Reference
	Rural	3.58 (2.37–5.41)	<0.0001	2.22 (1.15–4.31)	0.020
Children under 5	Continuous	4.89 (3.05–7.83)	<0.0001	1.77 (1.05–2.99)	0.034
Women of reproductive age	Continuous	1.21 (0.414–3.57)	0.714	1.00 (0.33–3.05)	0.999
ITN ownership	Continuous	4.30 (1.06–17.40)	0.042	2.15 (0.85–5.44)	0.102
Country-level spray coverage	<10%	Reference	Reference	Reference	Reference
	10–20%	0.44 (0.18–1.06)	0.065	0.55 (0.29–1.06)	0.073
	20–30%	4.26 (2.87–6.31)	<0.0001	4.70 (3.11–7.10)	<0.0001
	>30%	0.70 (0.23–2.13)	0.516	0.75 (0.27–2.13)	0.581

Standard errors were adjusted for correlated data at the dataset level

N = 2010 EAs, 30 datasets

## Discussion

### Key results

Household IRS coverage across sub-Saharan Africa has been typically low, with the majority of countries included in this analysis reporting <10% of households being sprayed. Given the expense of IRS operations compared to ITN distributions [7], and the uncertainty around the effectiveness of combining IRS with ITNs [10–12], this low coverage can be expected. Should further studies demonstrate an added benefit of IRS in the presence of ITNs, the household coverage would presumably increase. Additionally, with the spectre of pyrethroid resistance spreading across malaria-endemic regions there may be a need for malaria control programmes to consider IRS as a necessary vector control option [8, 9]. From the perspective of household coverage, IRS implementation appears to be rather good despite coverage being low across the continent. The majority of countries show equitable IRS coverage in terms of household wealth, i.e. IRS coverage favoring poorer households, and the majority of countries show equitable IRS coverage in terms of the urban/rural divide, i.e. IRS coverage favoring rural households where malaria transmission is inherently more intense.

Considering IRS coverage at the community level however suggests IRS implementation is incredibly poor across the continent. Effective spatial coverage is crucial for IRS, where a house protected with IRS does not necessarily prevent malaria transmission to that house but rather prevents malaria transmission from that house. For example, on Bioko Island houses receiving IRS in communities with <20% coverage saw no benefit to the intervention, but houses not receiving IRS in communities with ≥80% coverage saw the same benefit as their sprayed neighbours [14]. The literature on the relative effectiveness of increasing IRS coverage is sparse, and further studies need to be conducted to determine at what coverage level IRS investment would have more impact on malaria transmission if diverted to non-IRS interventions. Frustratingly, recent community-randomized controlled trials of IRS plus ITNs have not reported on spatial IRS coverage during implementation.

### Limitations

The analyses conducted herein were limited to publicly available two-stage cluster surveys that contained a measure of IRS coverage at the household level. Numerous malaria control programmes did not have a survey conducted during this time period, or had only one survey conducted. The results of poor community-level coverage must be taken with caution for a few reasons. First, as indicated previously the specific questions referring to IRS in the surveys may be misinterpreted leading to great misclassification error. In limiting the community-level coverage analysis to clusters where at least 5 houses reported being covered by IRS, these errors are expected to have limited influence on the analysis. Second, these analyses do not take into account programme data in defining the areas where large IRS programmes have been conducted. The use of non-programme data to evaluate coverage was required given that IRS programmes currently do not measure coverage as relative to actual population but rather use the number of houses found by spray operators as the denominator in their coverage indicator (Bridges et al., pers. comm.). Still, clusters analysed herein may have only partially been targeted for IRS coverage, if at all, which would bias the results toward poor community-level coverage.

### Interpretation

The issues found in IRS implementation in these analyses are not unique to IRS nor malaria. Implementing any public health intervention at a community-level coverage >85% is challenging, particularly in environments with limited spatial information on house locations and population numbers. Similar coverage challenges were observed in polio campaigns in Nigeria where large portions of human settlements were missed by vaccination teams operating without a clear geospatial picture [17]. Coverage increased greatly when the polio vaccine campaigns began using satellite imagery and geospatial monitoring to both define areas that needed vaccines and identify where vaccine teams had and had not reached [18, 19]. Similar tools have been developed for IRS [20, 21], and these tools have been associated with improving IRS coverage in both Zambia (Bridges et al., pers. comm.) and Equatorial Guinea [22].

## Conclusion

Although IRS implementation typically targets poorer, rural households, spatial coverage achieved is quite poor across sub-Saharan Africa. Better indicators and improved monitoring are needed to ensure that high coverage is achieved and resources invested in IRS are maximized.

## Additional files

**Additional file 1.** LQAS decision points used in the analysis.

**Additional file 2.** Map showing IRS coverage by community in Angola 2010–2011.

**Additional file 3.** Map showing IRS coverage by community in Benin 2012.

**Additional file 4.** Map showing IRS coverage by community in Burkina Faso 2010.

**Additional file 5.** Map showing IRS coverage by community in Burkina Faso 2014.

**Additional file 6.** Map showing IRS coverage by community in Cameroon 2011.

**Additional file 7.** Map showing IRS coverage by community in Cote d’Ivoire 2012.

**Additional file 8.** Map showing IRS coverage by community in Gabon 2012.

**Additional file 9.** Map showing IRS coverage by community in Ghana 2014.

**Additional file 10.** Map showing IRS coverage by community in Guinea 2012.

**Additional file 11.** Map showing IRS coverage by community in Kenya 2014.

**Additional file 12.** Map showing IRS coverage by community in Liberia 2011.

**Additional file 13.** Map showing IRS coverage by community in Liberia 2013.

**Additional file 14.** Map showing IRS coverage by community in Madagascar 2011.

**Additional file 15.** Map showing IRS coverage by community in Madagascar 2013.

**Additional file 16.** Map showing IRS coverage by community in Malawi 2010.

**Additional file 17.** Map showing IRS coverage by community in Malawi 2012.

**Additional file 18.** Map showing IRS coverage by community in Malawi 2014.

**Additional file 19.** Map showing IRS coverage by community in Mali 2012–2013.

**Additional file 20.** Map showing IRS coverage by community in Mozambique 2011.

**Additional file 21.** Map showing IRS coverage by community in Namibia 2013.

**Additional file 22.** Map showing IRS coverage by community in Nigeria 2010.

**Additional file 23.** Map showing IRS coverage by community in Nigeria 2013.

**Additional file 24.** Map showing IRS coverage by community in Tanzania 2011.

**Additional file 25.** Map showing IRS coverage by community in Uganda 2011.

**Additional file 26.** Map showing IRS coverage by community in Uganda 2014–2015.

**Additional file 27.** Map showing IRS coverage by community in Zambia 2013.

**Additional file 28.** Map showing IRS coverage by community in Zimbabwe 2010–2011.

## Abbreviations

AIS: AIDS indicator survey
DHS: demographic and health survey
IRS: indoor residual spray
ITN: insecticide-treated mosquito net
LQAS: lot-quality assurance sampling
MICS: multiple indicator cluster survey
MIS: malaria indicator survey
PMI: President’s Malaria Initiative
