# Supplementary figures and images for: Reported community-level indoor residual spray coverage from two-stage cluster surveys in sub-Saharan Africa
Source: Malar J. 2017 Jun 13;16:249. doi: 10.1186/s12936-017-1893-x (PMC5470197; doi:10.1186/s12936-017-1893-x)

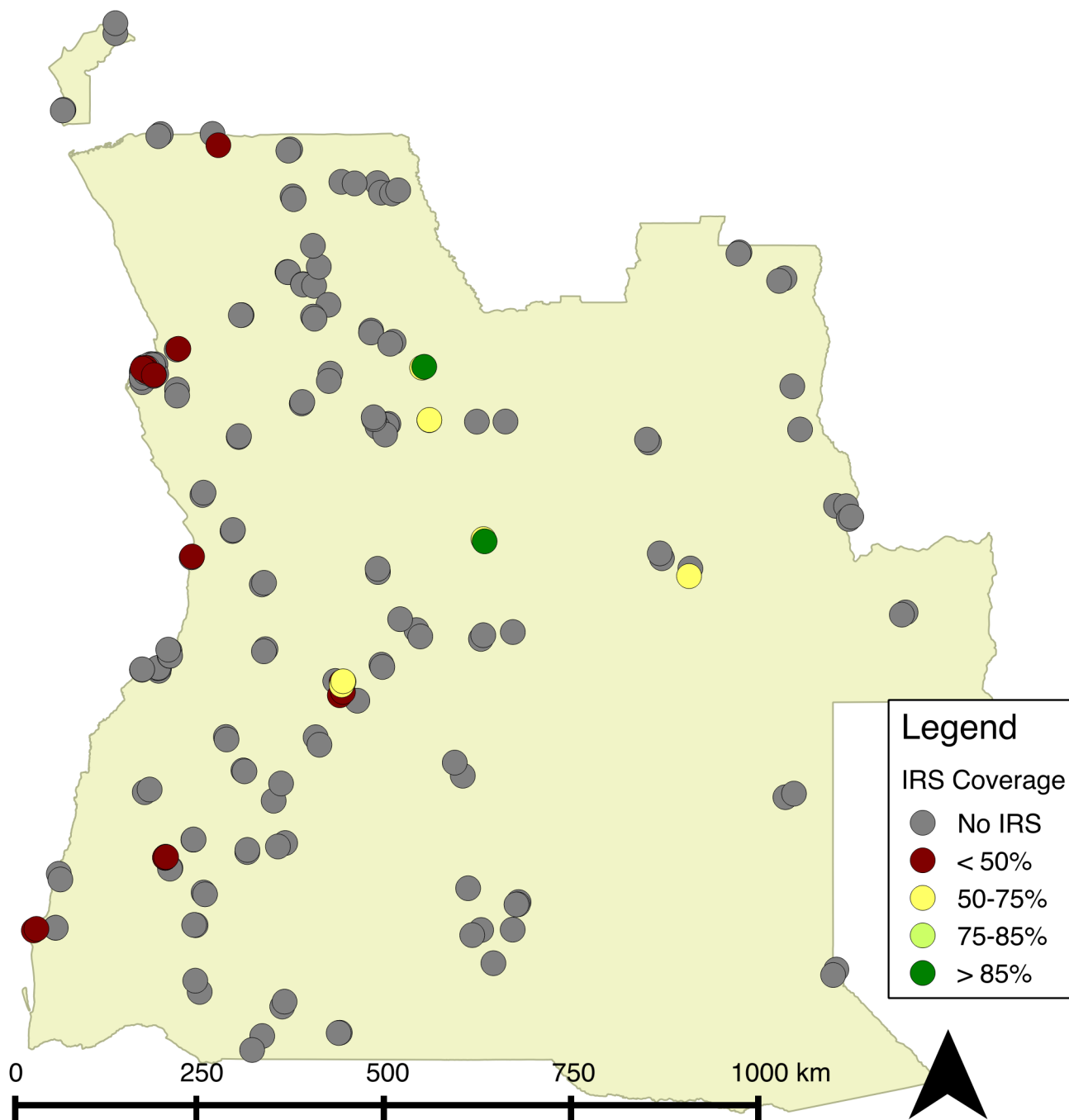

Supplement: Supplementary file 2 — Additional file 2. Map showing IRS coverage by community in Angola 2010–2011. [file 12936_2017_1893_MOESM2_ESM.pdf]

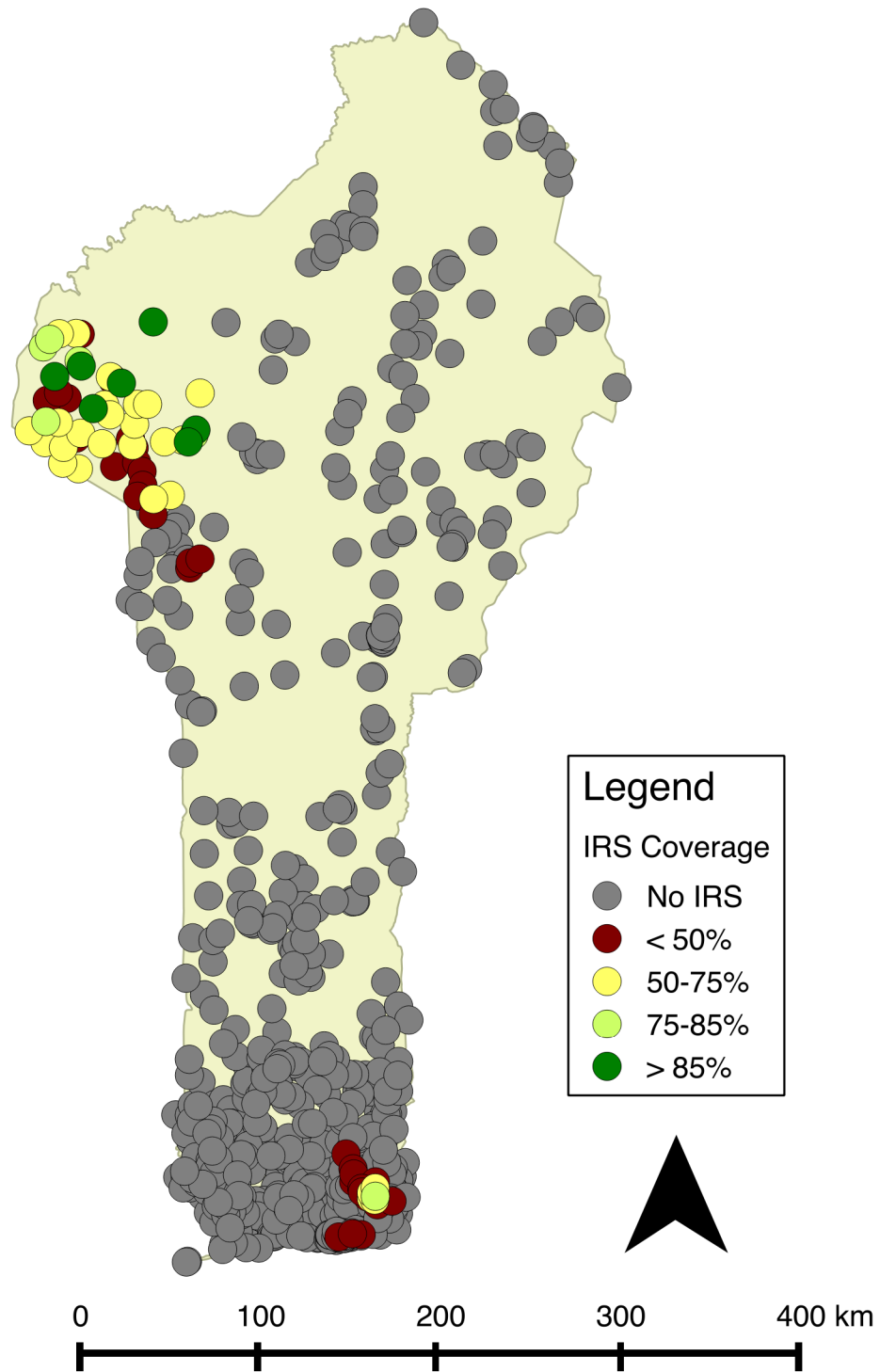

Supplement: Supplementary file 3 — Additional file 3. Map showing IRS coverage by community in Benin 2012. [file 12936_2017_1893_MOESM3_ESM.pdf]

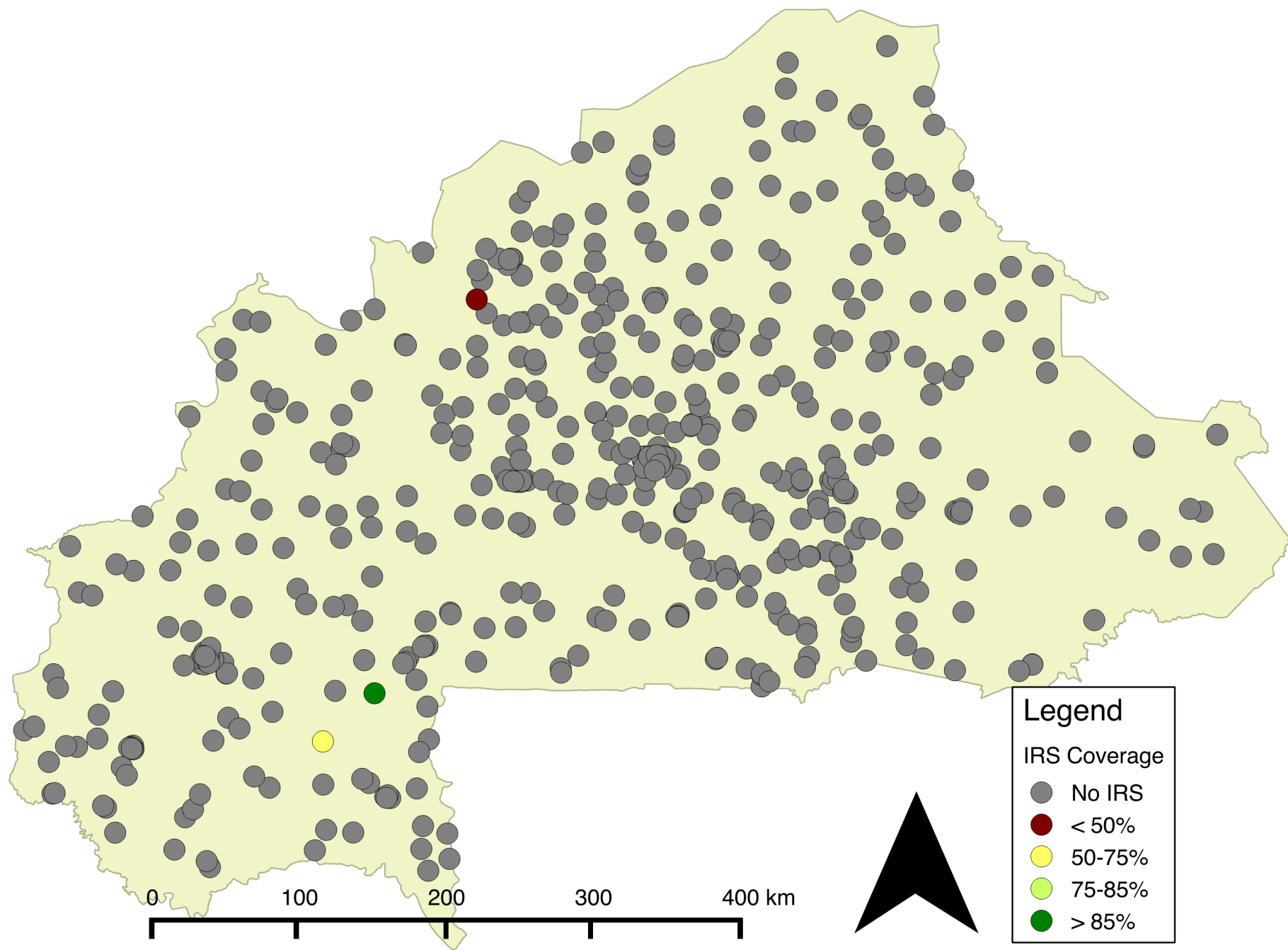

Supplement: Supplementary file 4 — Additional file 4. Map showing IRS coverage by community in Burkina Faso 2010. [file 12936_2017_1893_MOESM4_ESM.pdf]

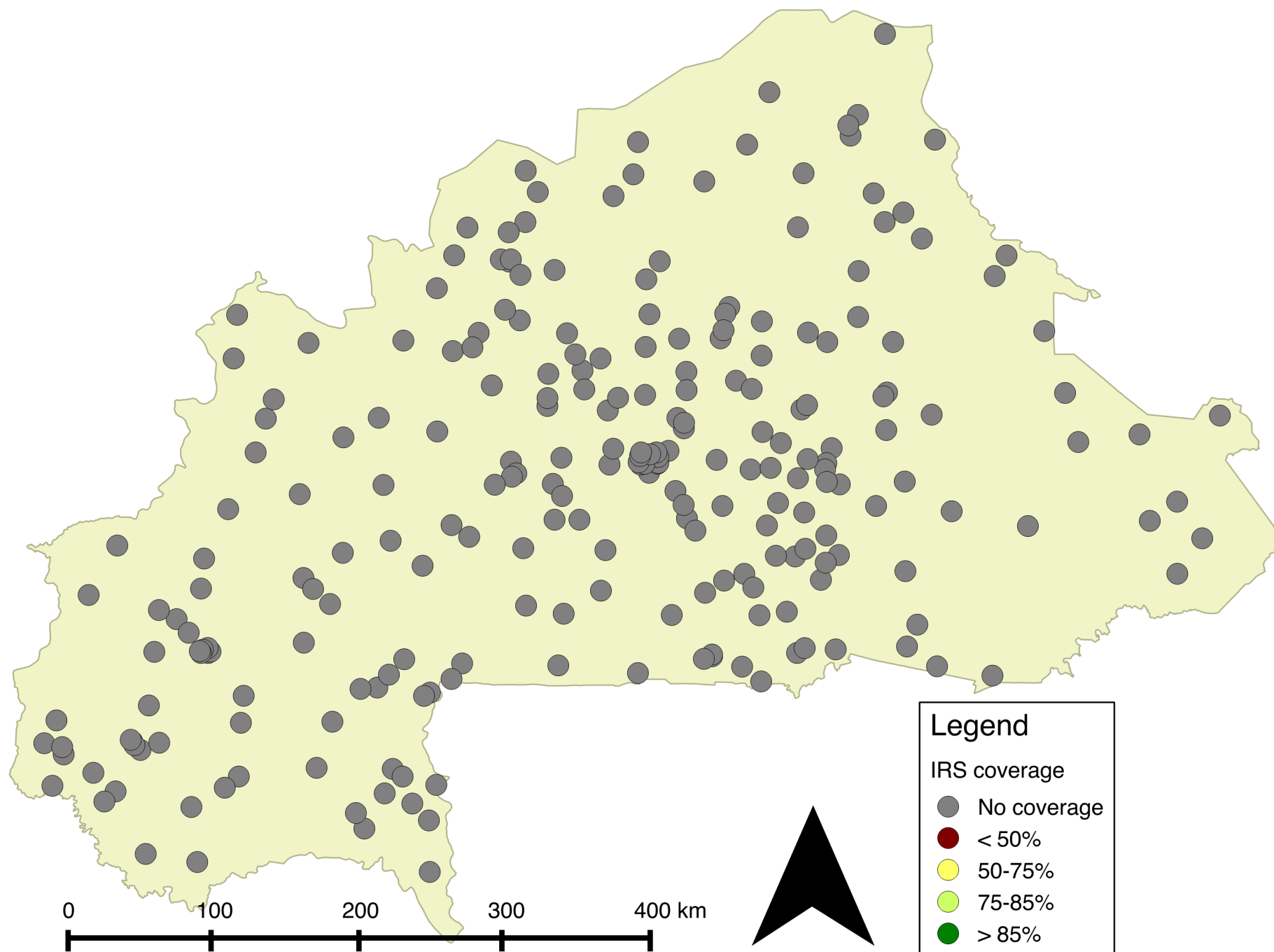

Supplement: Supplementary file 5 — Additional file 5. Map showing IRS coverage by community in Burkina Faso 2014. [file 12936_2017_1893_MOESM5_ESM.pdf]

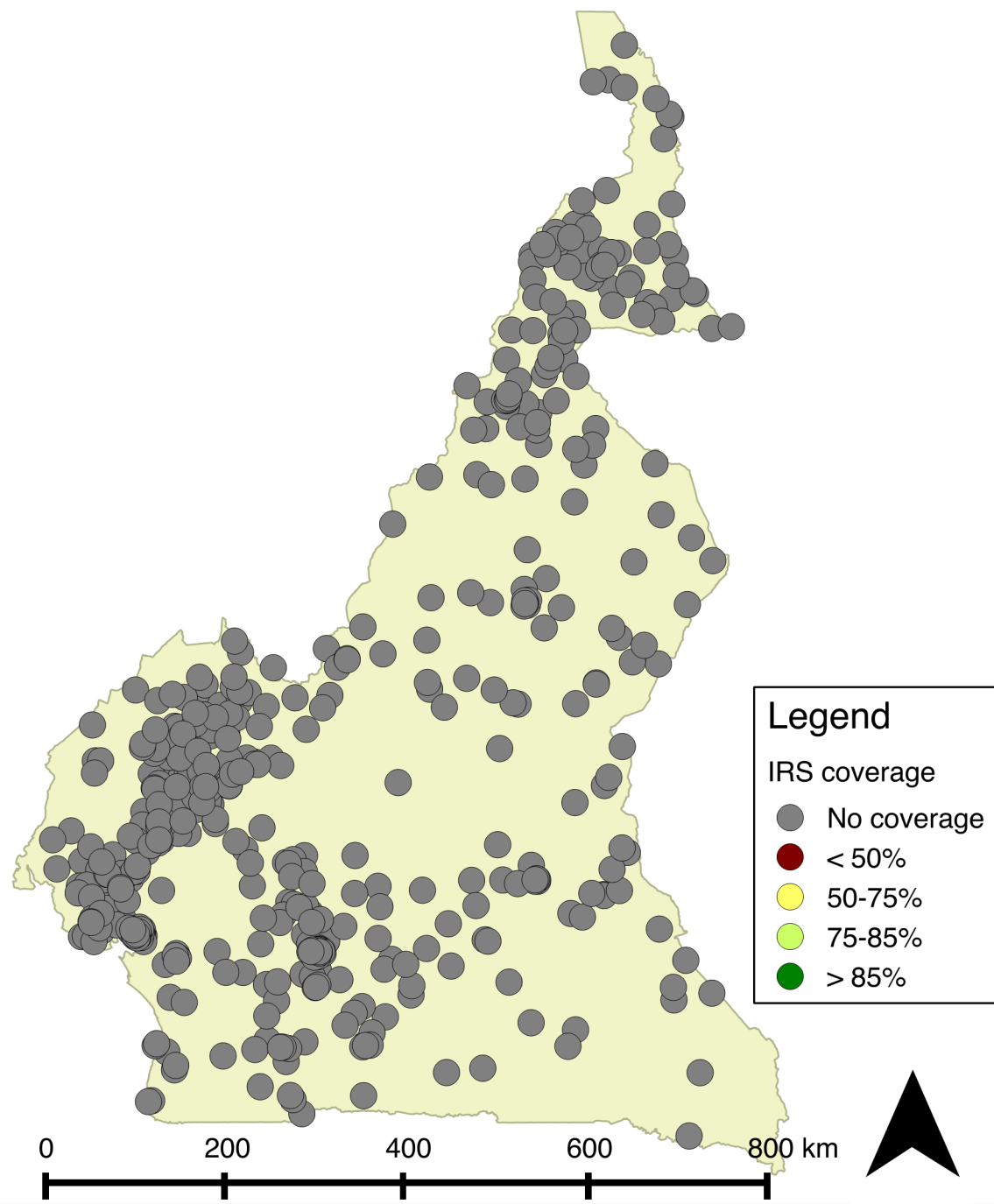

Supplement: Supplementary file 6 — Additional file 6. Map showing IRS coverage by community in Cameroon 2011. [file 12936_2017_1893_MOESM6_ESM.pdf]

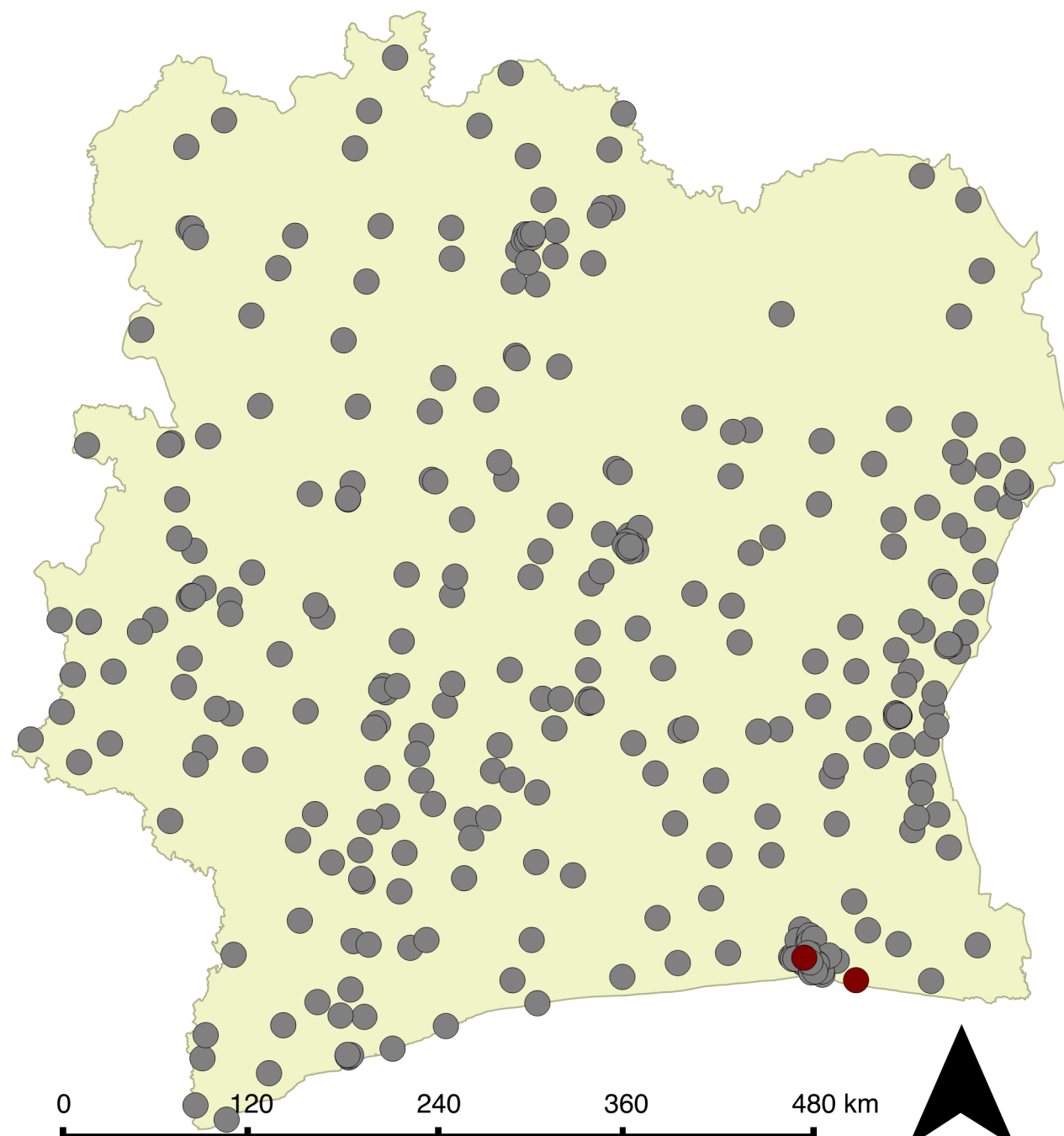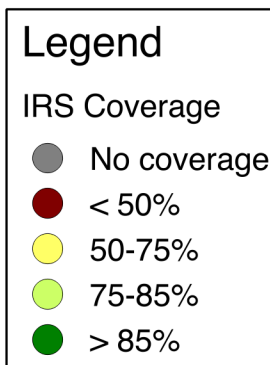

Supplement: Supplementary file 7 — Additional file 7. Map showing IRS coverage by community in Cote d’Ivoire 2012. [file 12936_2017_1893_MOESM7_ESM.pdf]

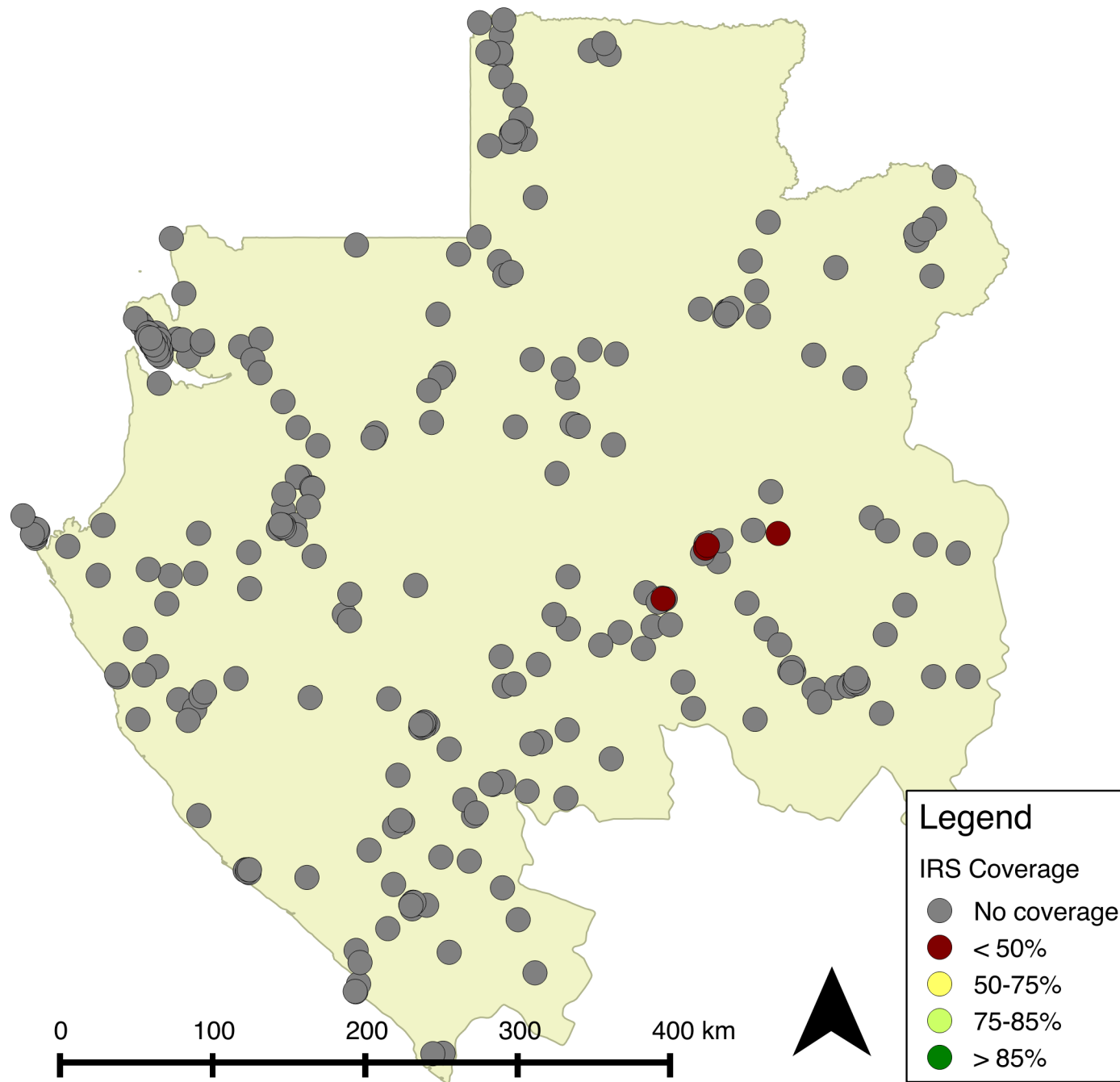

Supplement: Supplementary file 8 — Additional file 8. Map showing IRS coverage by community in Gabon 2012. [file 12936_2017_1893_MOESM8_ESM.pdf]

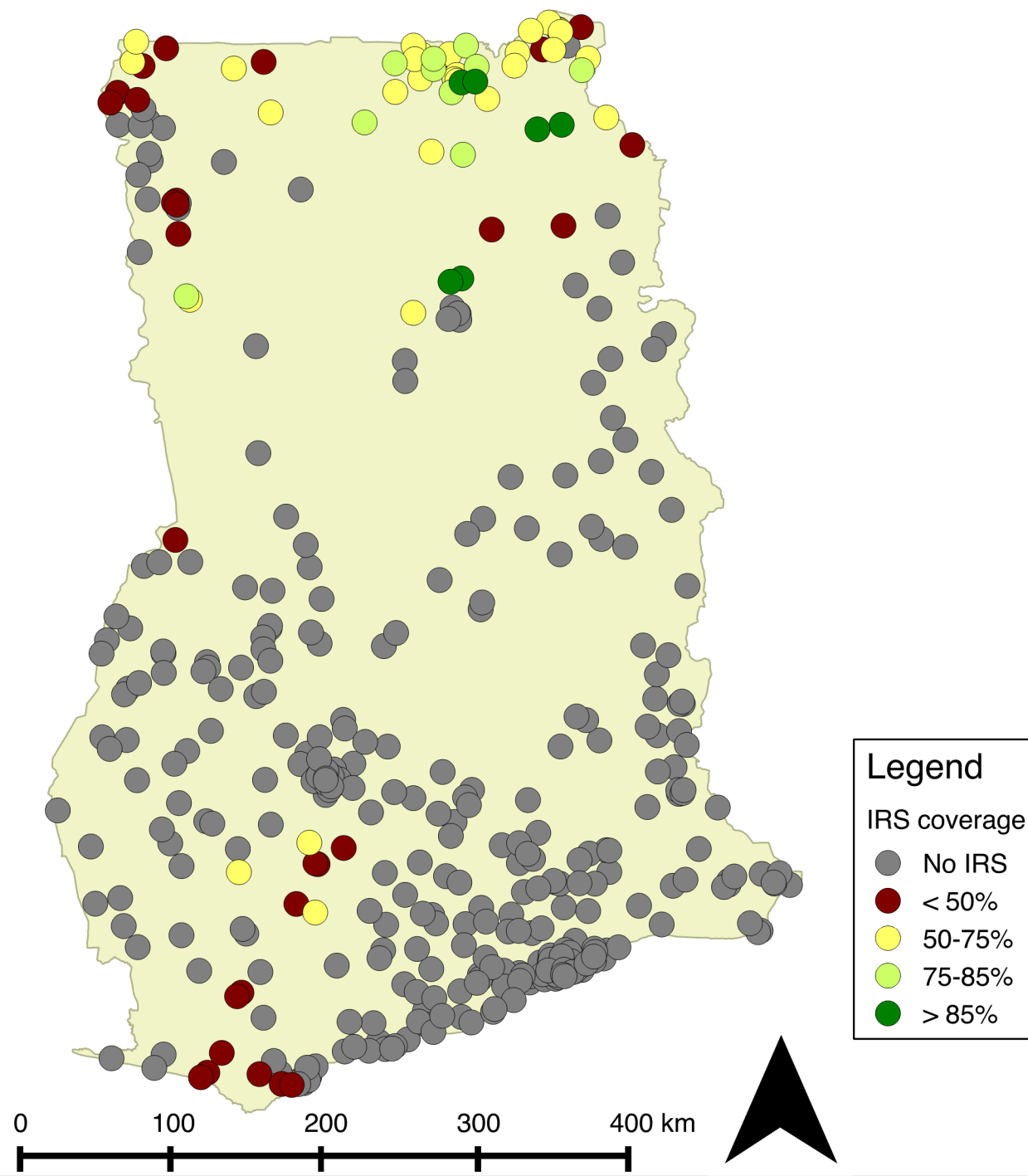

Supplement: Supplementary file 9 — Additional file 9. Map showing IRS coverage by community in Ghana 2014. [file 12936_2017_1893_MOESM9_ESM.pdf]

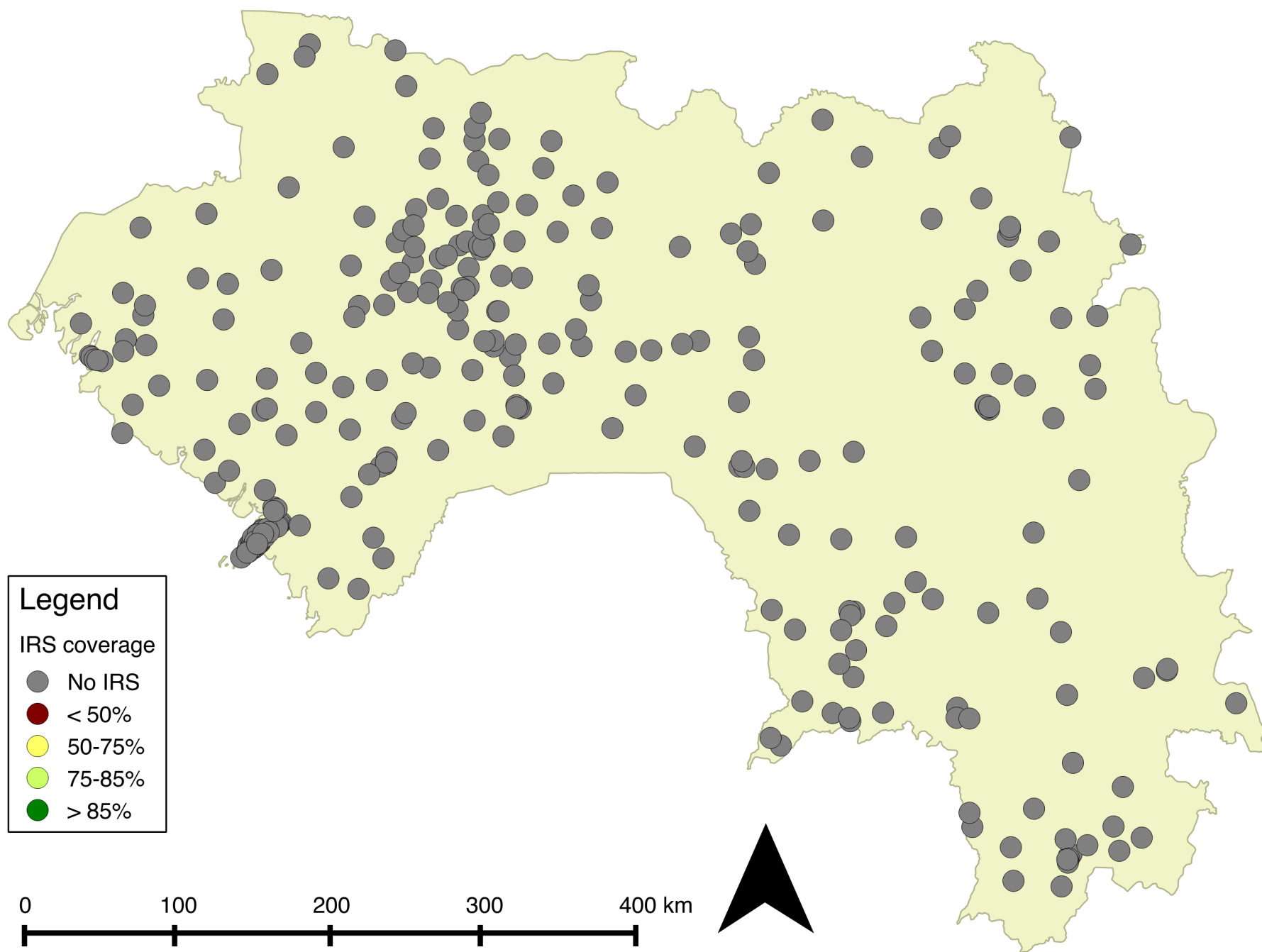

Supplement: Supplementary file 10 — Additional file 10. Map showing IRS coverage by community in Guinea 2012. [file 12936_2017_1893_MOESM10_ESM.pdf]

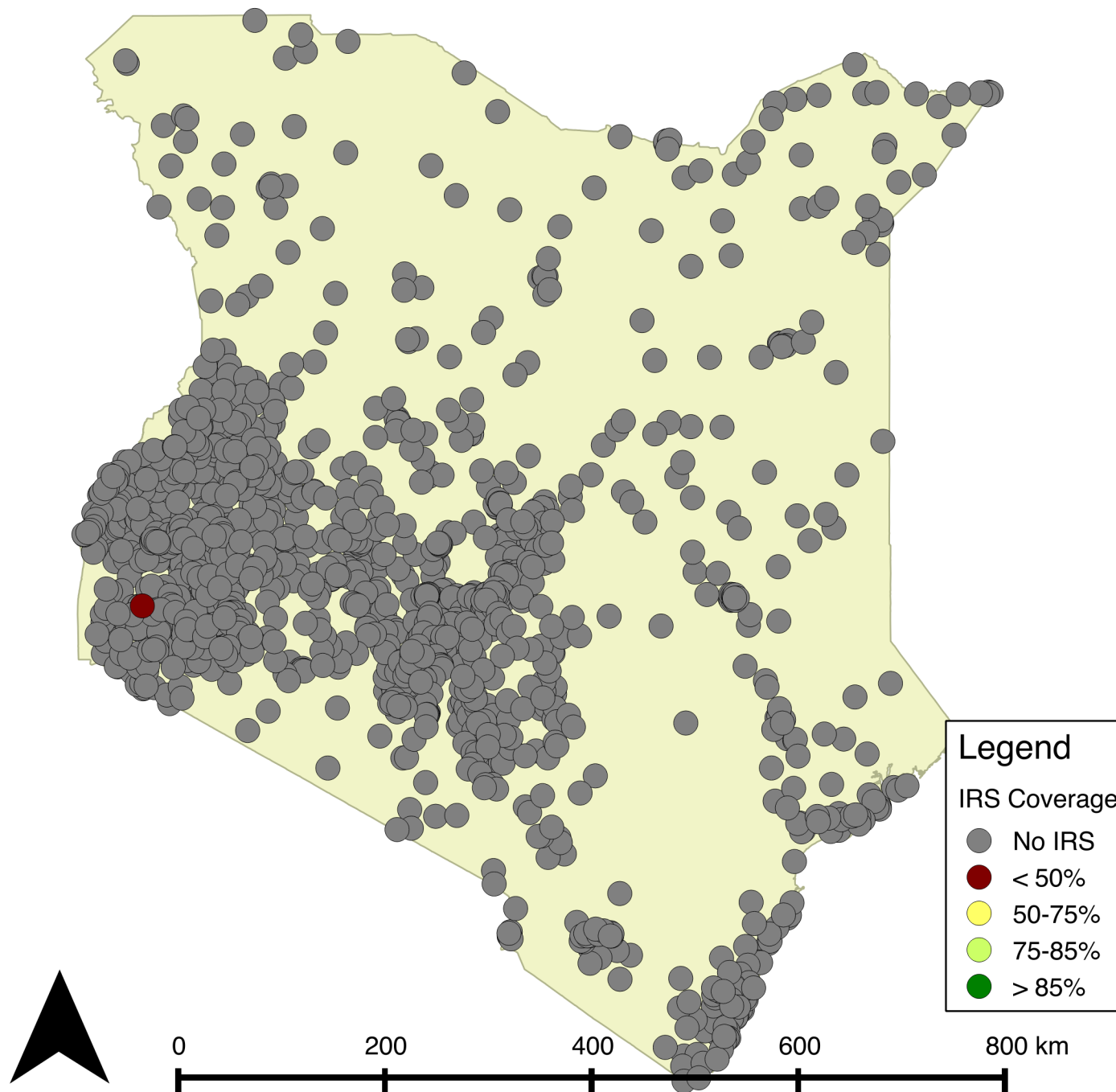

Supplement: Supplementary file 11 — Additional file 11. Map showing IRS coverage by community in Kenya 2014. [file 12936_2017_1893_MOESM11_ESM.pdf]

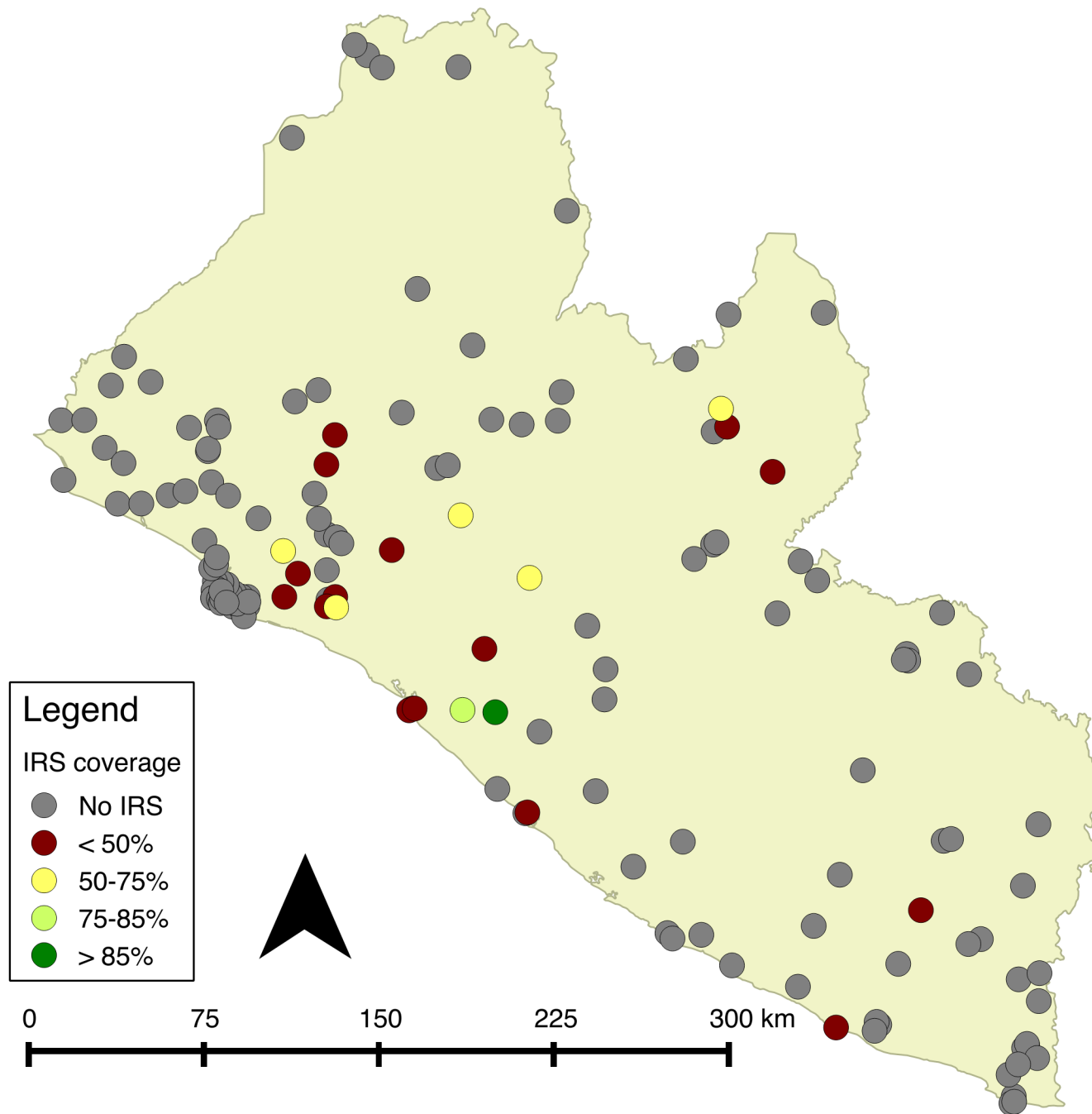

Supplement: Supplementary file 12 — Additional file 12. Map showing IRS coverage by community in Liberia 2011. [file 12936_2017_1893_MOESM12_ESM.pdf]

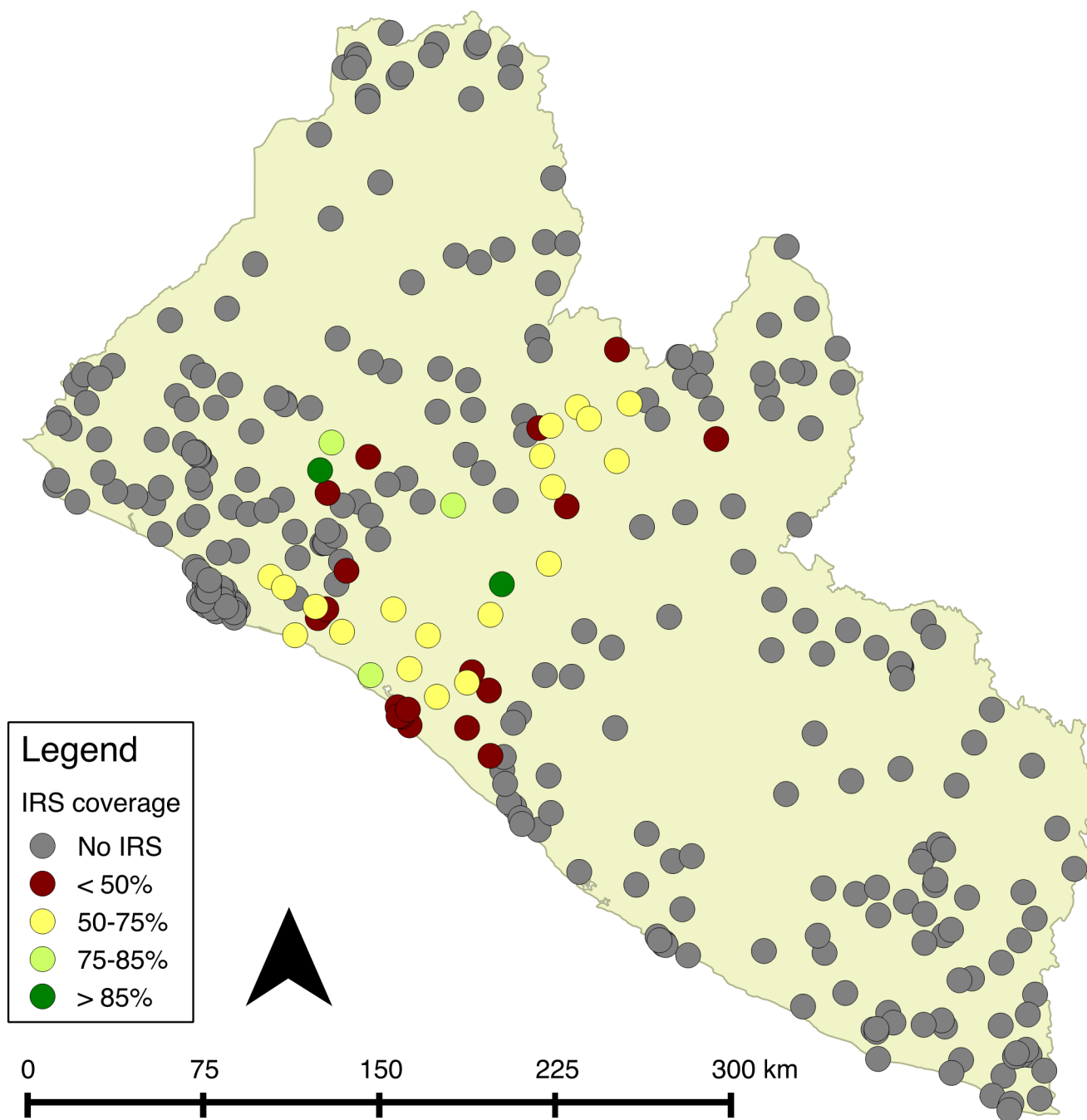

Supplement: Supplementary file 13 — Additional file 13. Map showing IRS coverage by community in Liberia 2013. [file 12936_2017_1893_MOESM13_ESM.pdf]

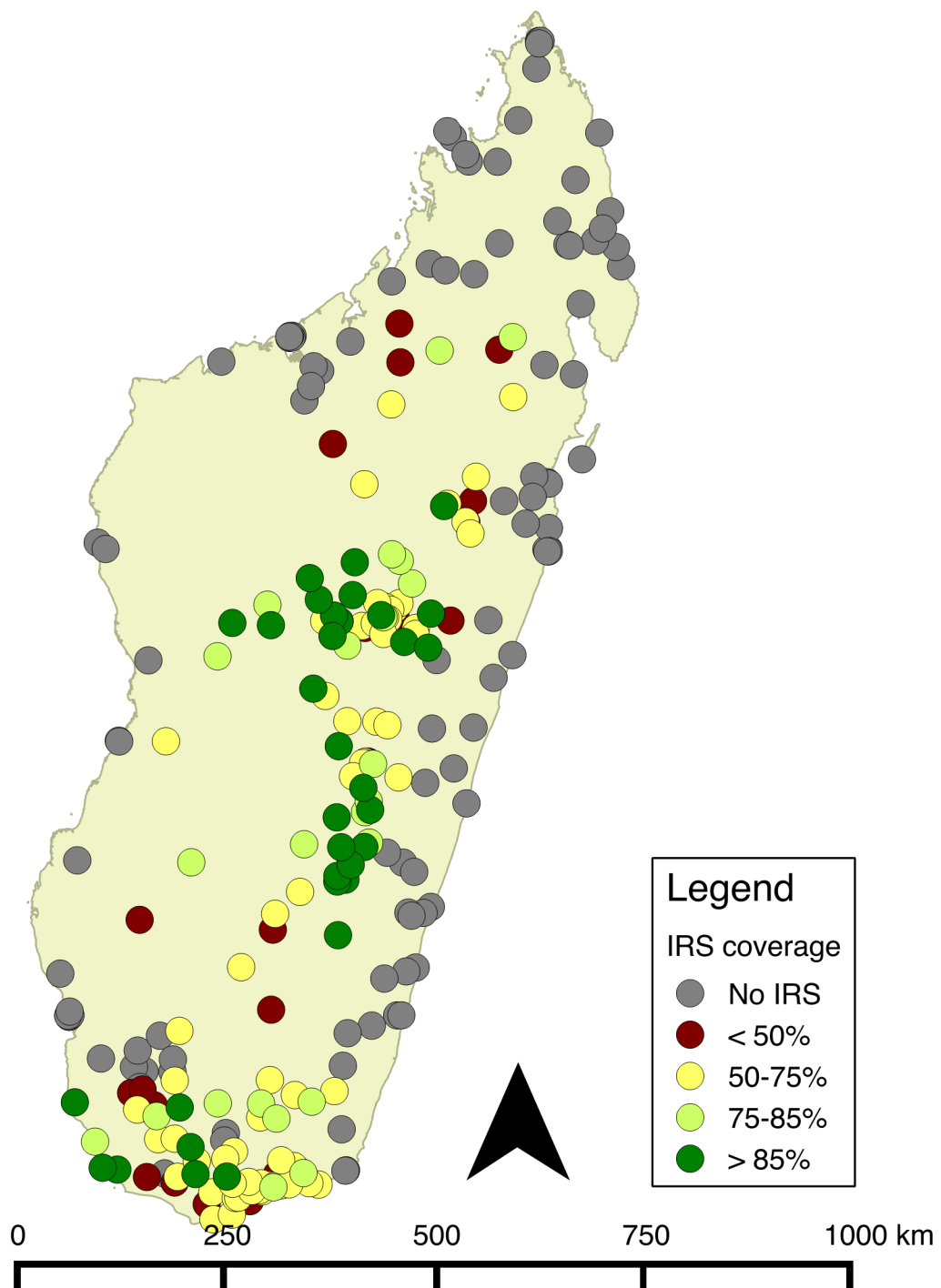

Supplement: Supplementary file 14 — Additional file 14. Map showing IRS coverage by community in Madagascar 2011. [file 12936_2017_1893_MOESM14_ESM.pdf]

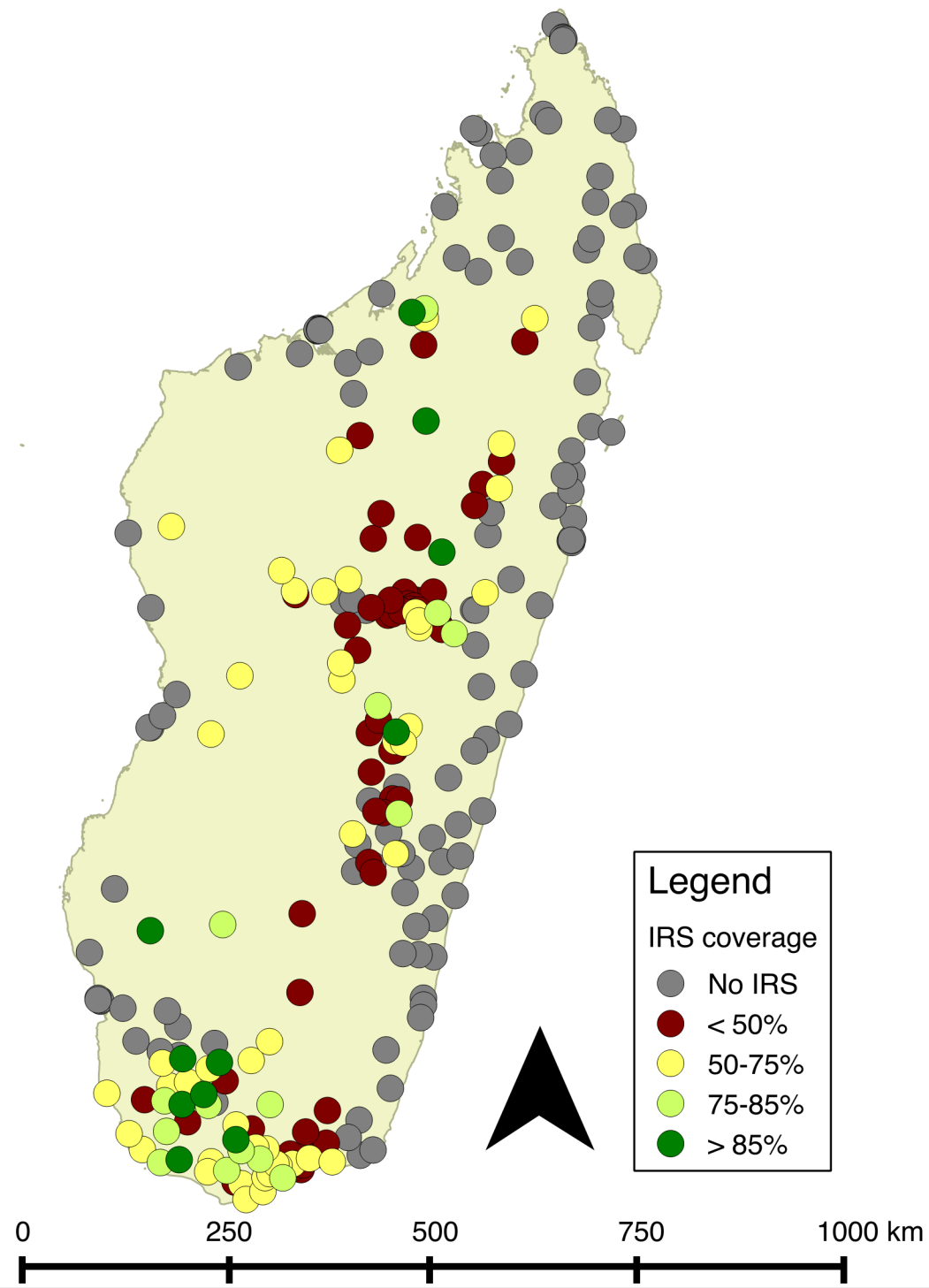

Supplement: Supplementary file 15 — Additional file 15. Map showing IRS coverage by community in Madagascar 2013. [file 12936_2017_1893_MOESM15_ESM.pdf]

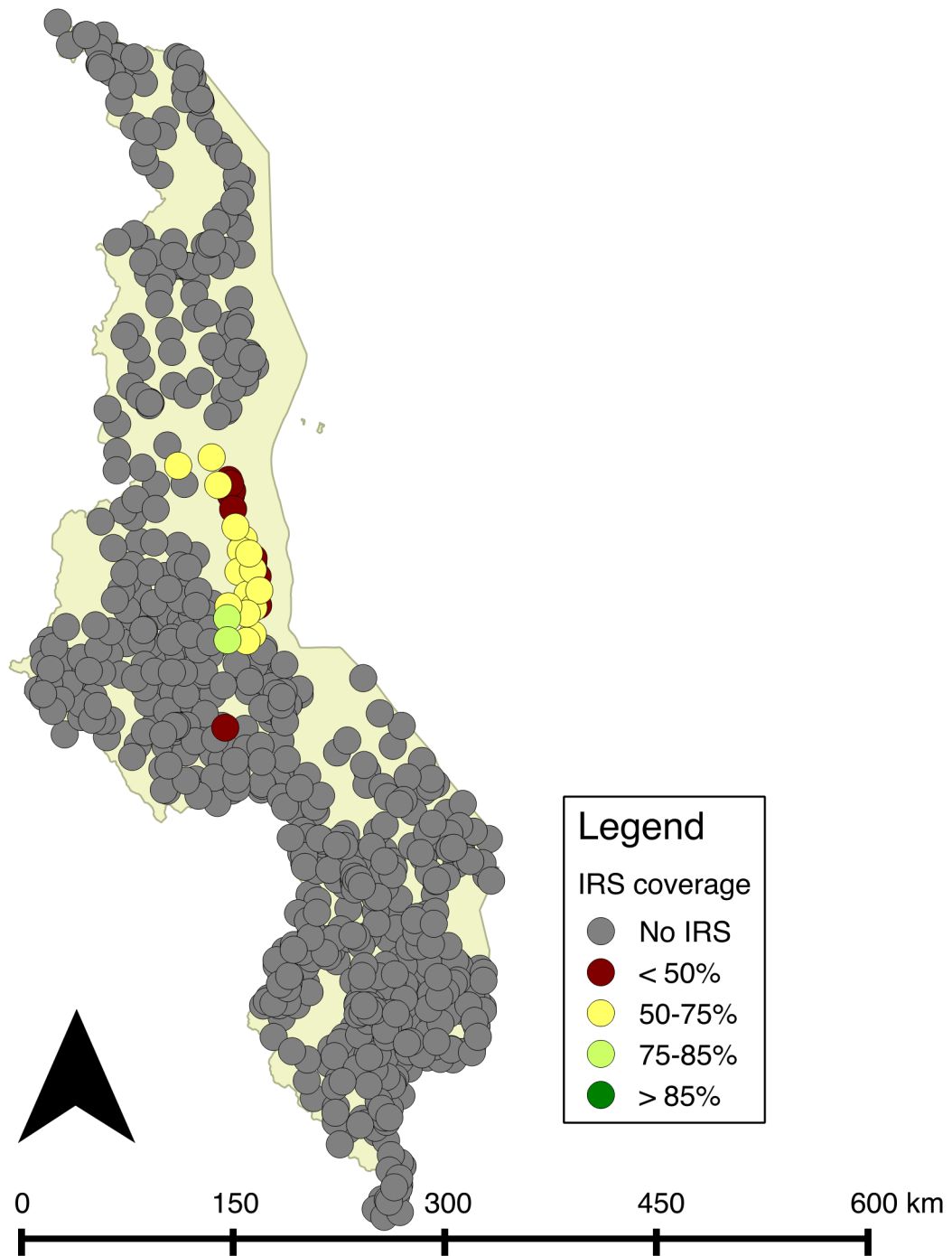

Supplement: Supplementary file 16 — Additional file 16. Map showing IRS coverage by community in Malawi 2010. [file 12936_2017_1893_MOESM16_ESM.pdf]

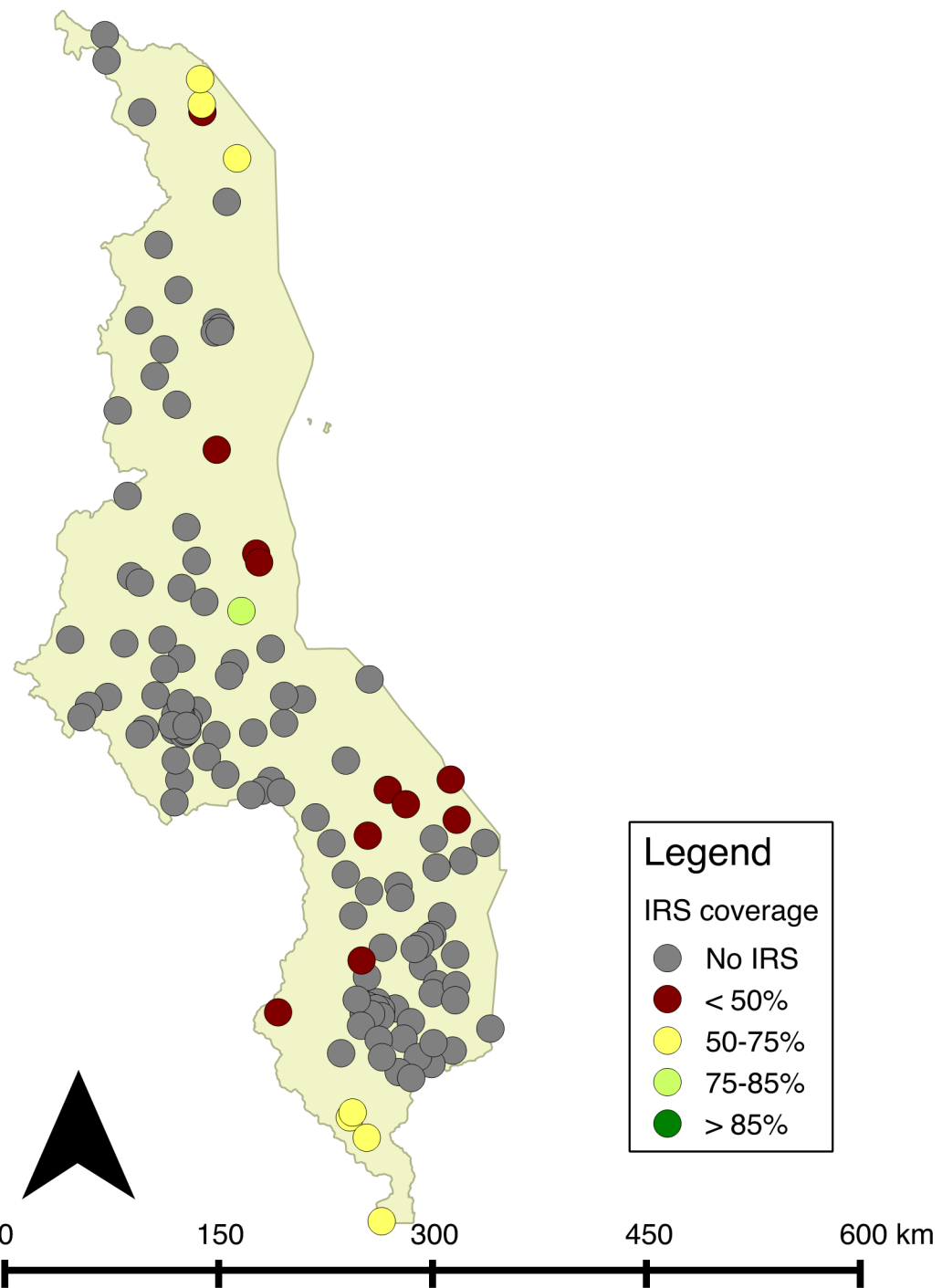

Supplement: Supplementary file 17 — Additional file 17. Map showing IRS coverage by community in Malawi 2012. [file 12936_2017_1893_MOESM17_ESM.pdf]

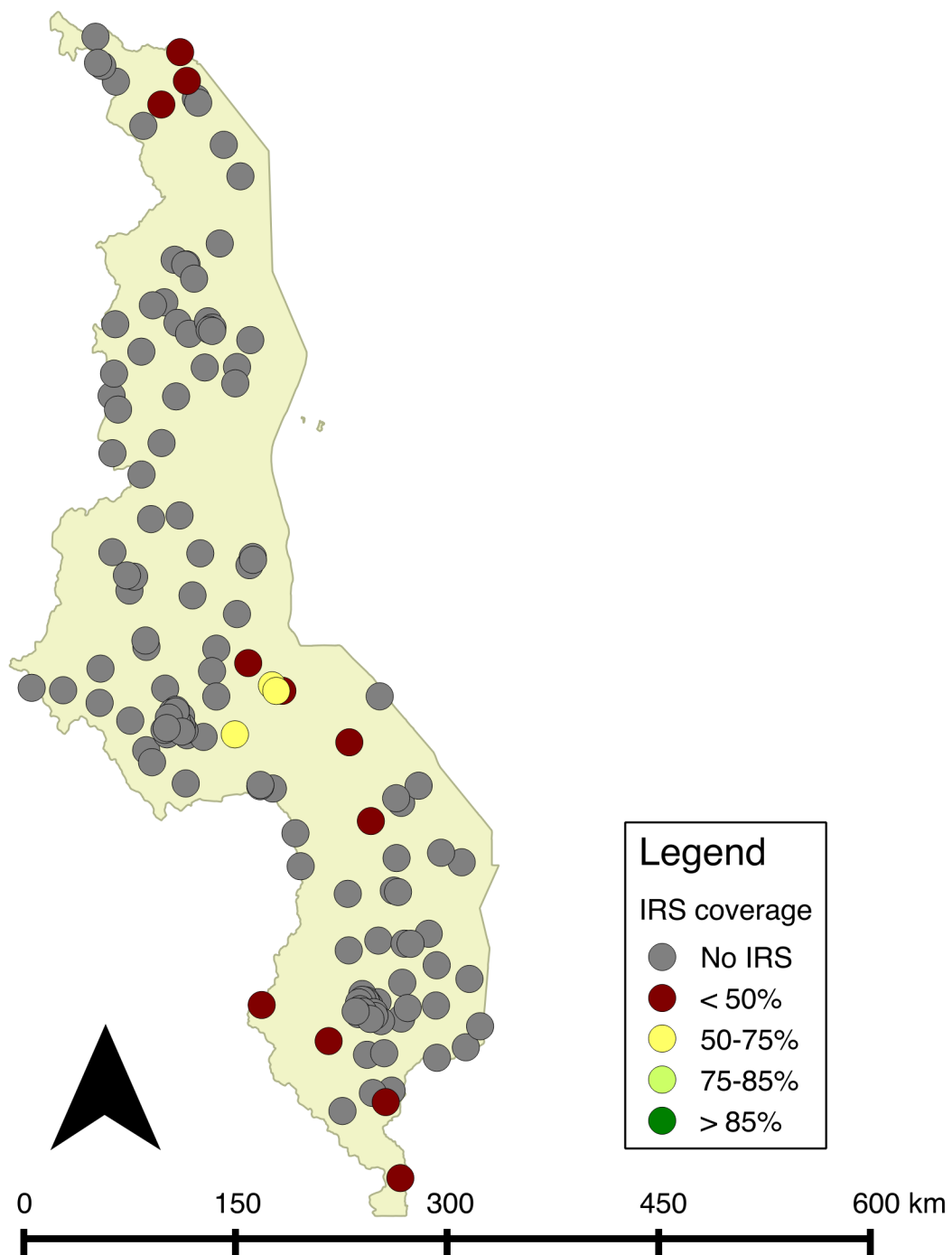

Supplement: Supplementary file 18 — Additional file 18. Map showing IRS coverage by community in Malawi 2014. [file 12936_2017_1893_MOESM18_ESM.pdf]

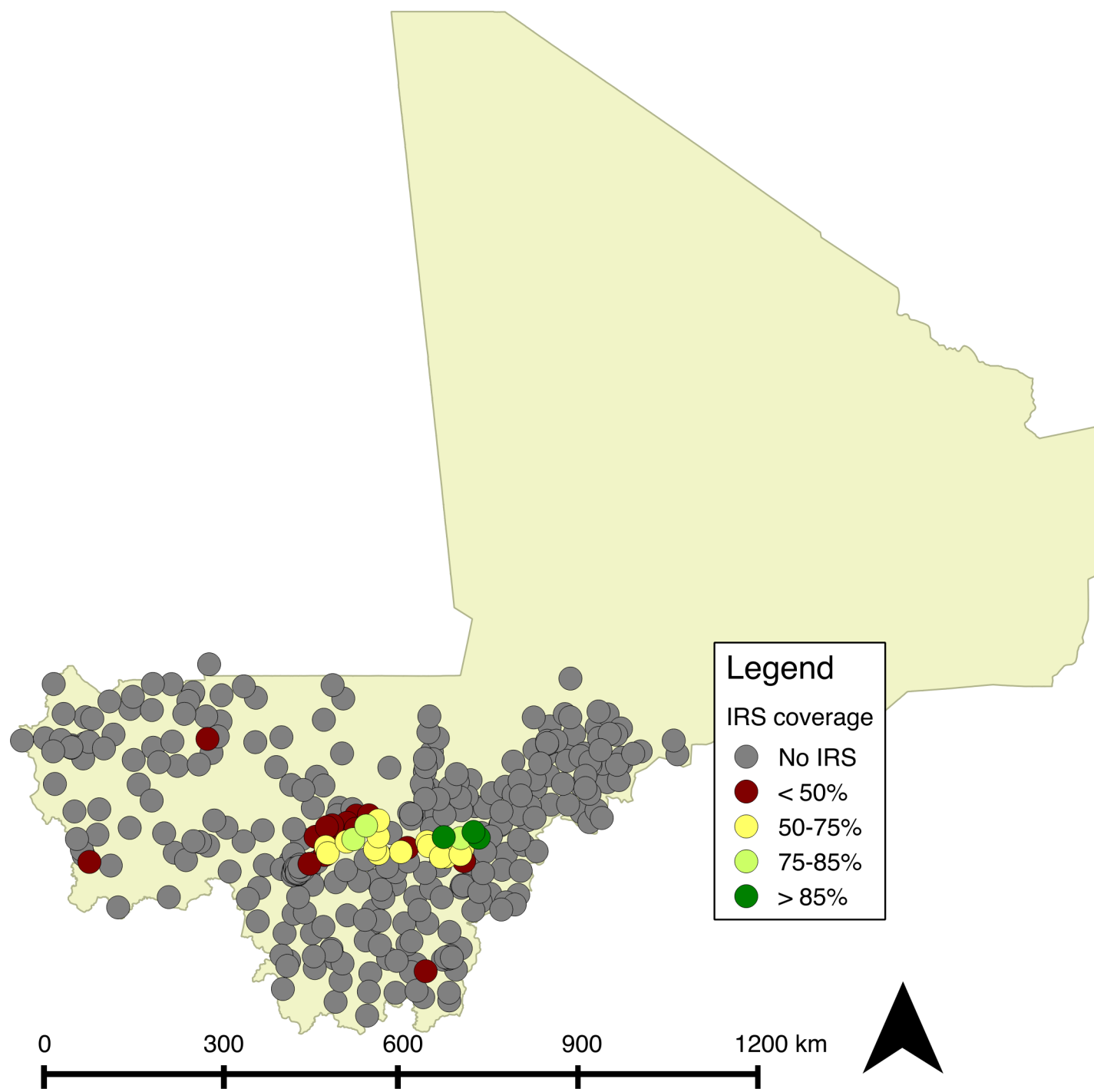

Supplement: Supplementary file 19 — Additional file 19. Map showing IRS coverage by community in Mali 2012–2013. [file 12936_2017_1893_MOESM19_ESM.pdf]

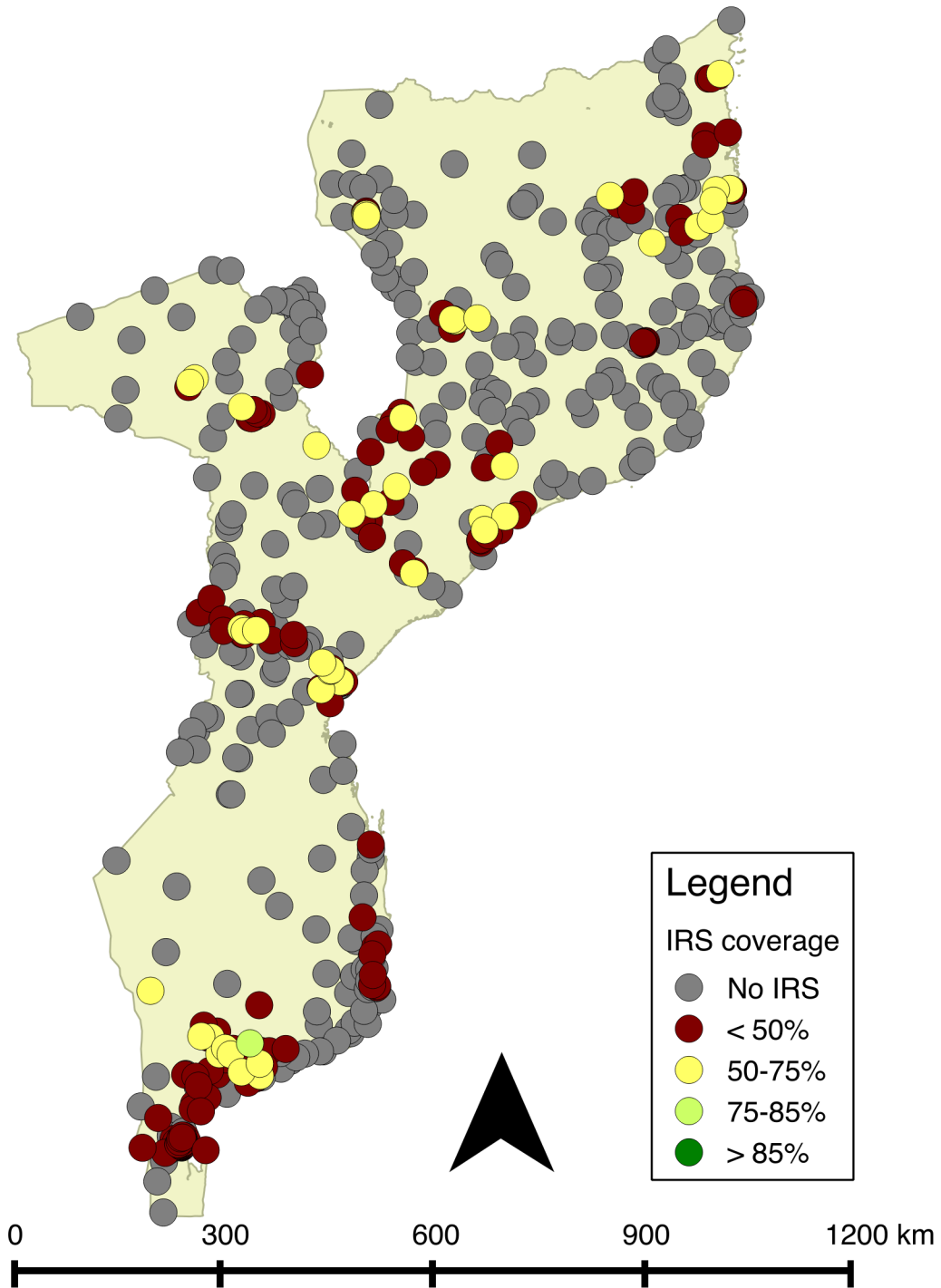

Supplement: Supplementary file 20 — Additional file 20. Map showing IRS coverage by community in Mozambique 2011. [file 12936_2017_1893_MOESM20_ESM.pdf]

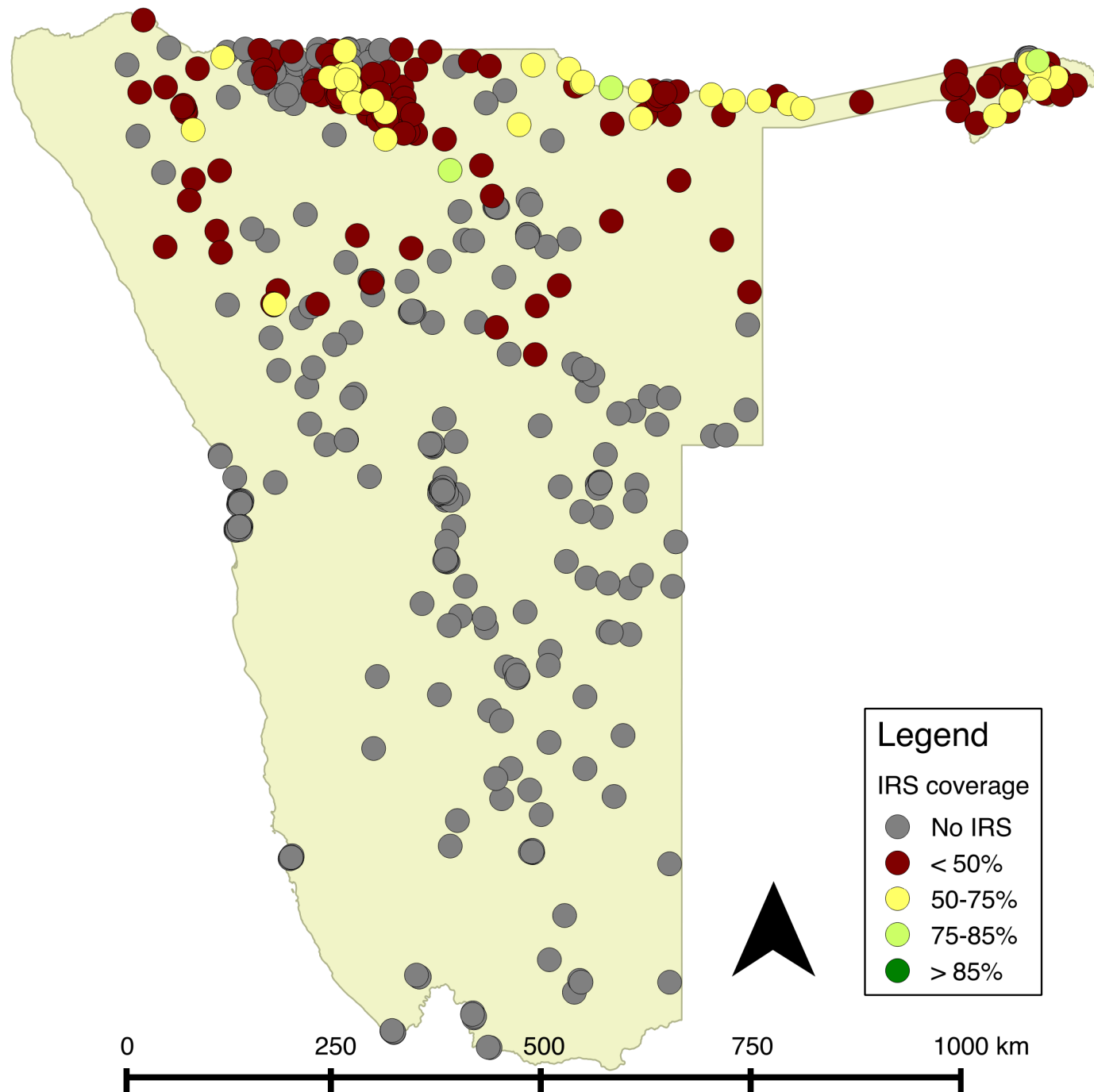

Supplement: Supplementary file 21 — Additional file 21. Map showing IRS coverage by community in Namibia 2013. [file 12936_2017_1893_MOESM21_ESM.pdf]

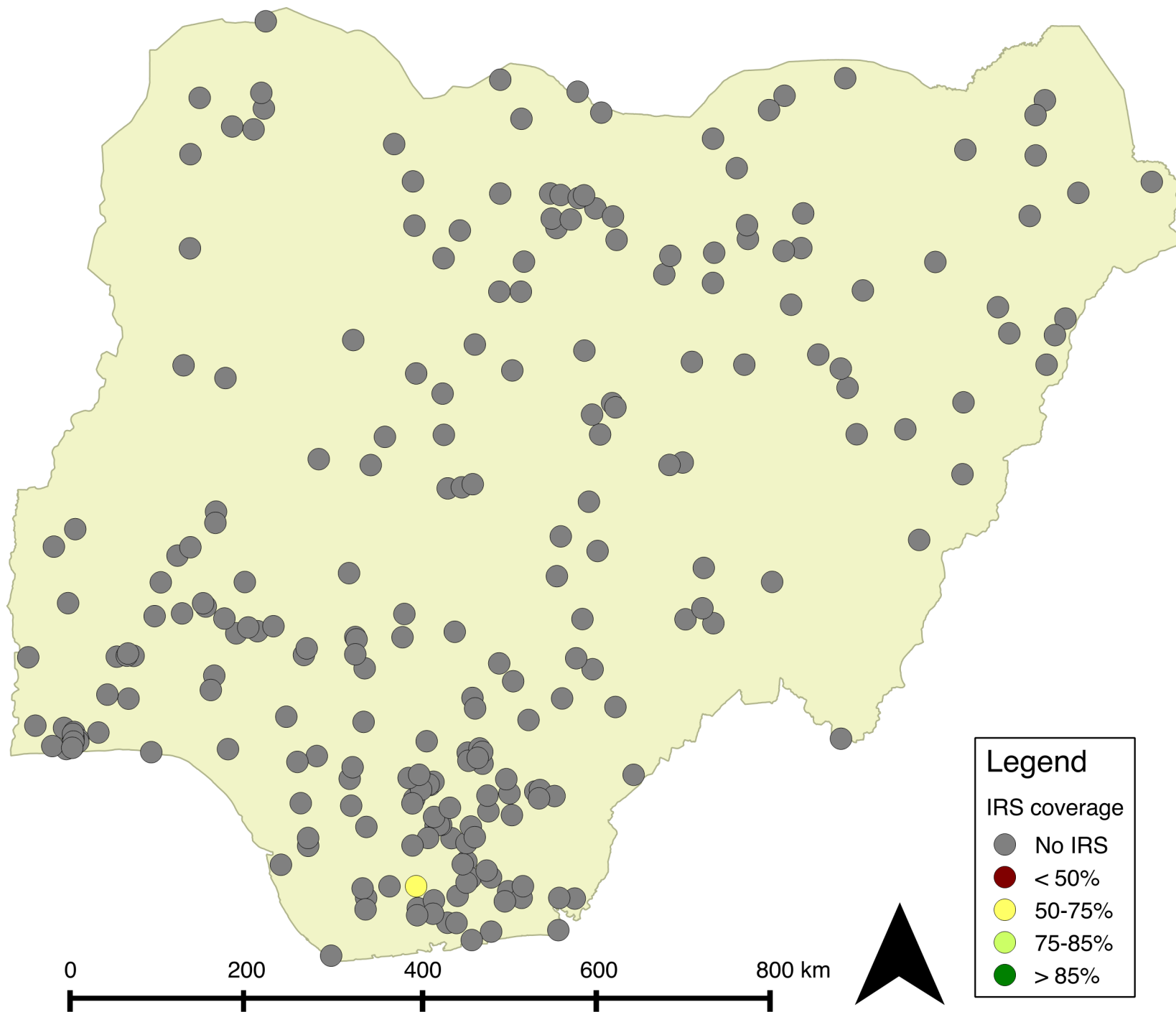

Supplement: Supplementary file 22 — Additional file 22. Map showing IRS coverage by community in Nigeria 2010. [file 12936_2017_1893_MOESM22_ESM.pdf]

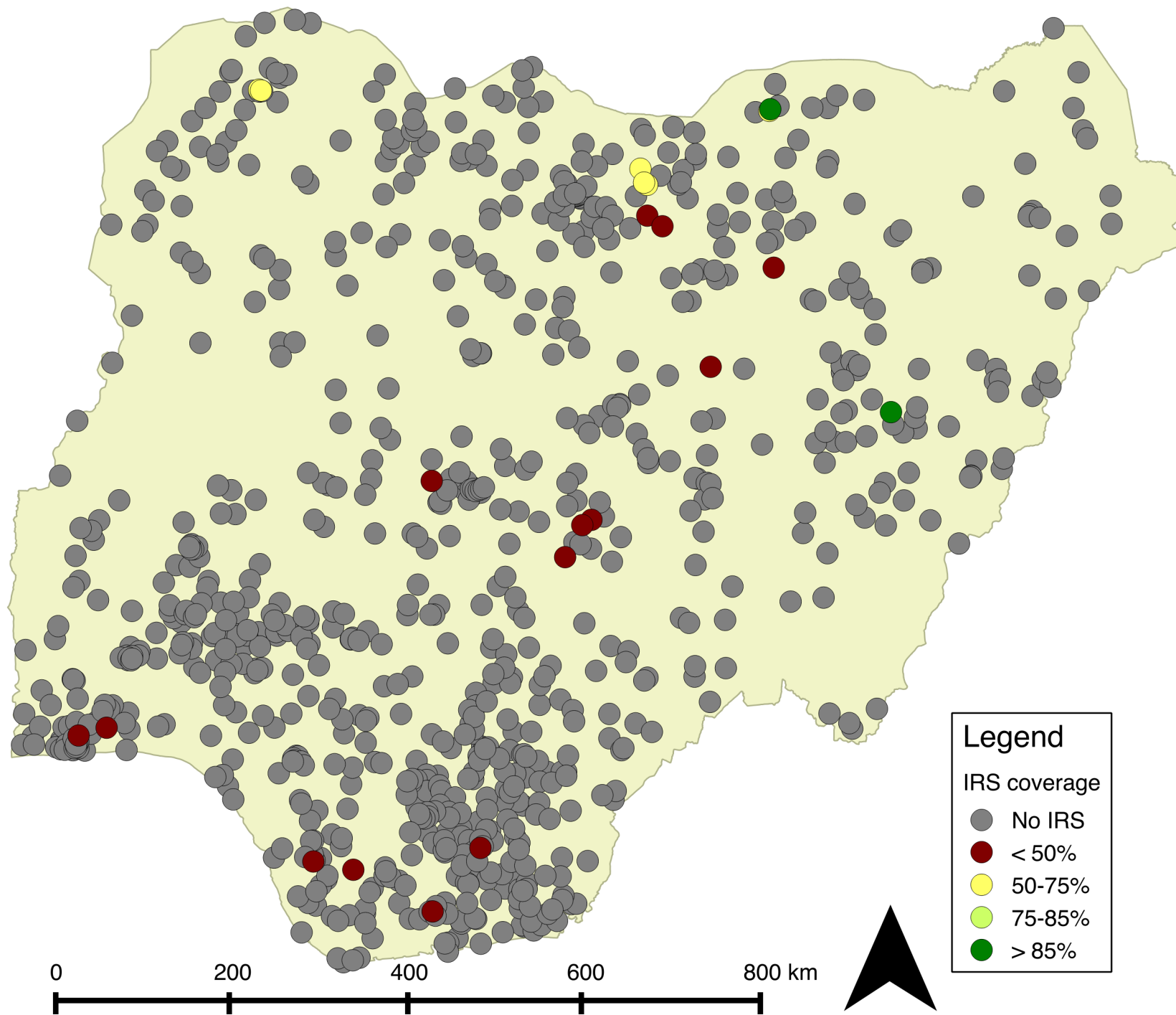

**Legend**

IRS coverage

- No IRS
- < 50%
- 50-75%
- 75-85%
- > 85%

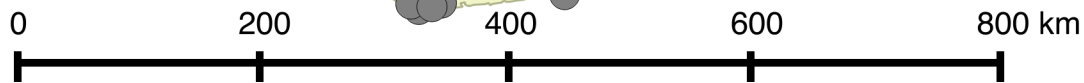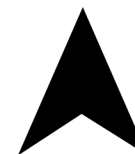

Supplement: Supplementary file 23 — Additional file 23. Map showing IRS coverage by community in Nigeria 2013. [file 12936_2017_1893_MOESM23_ESM.pdf]

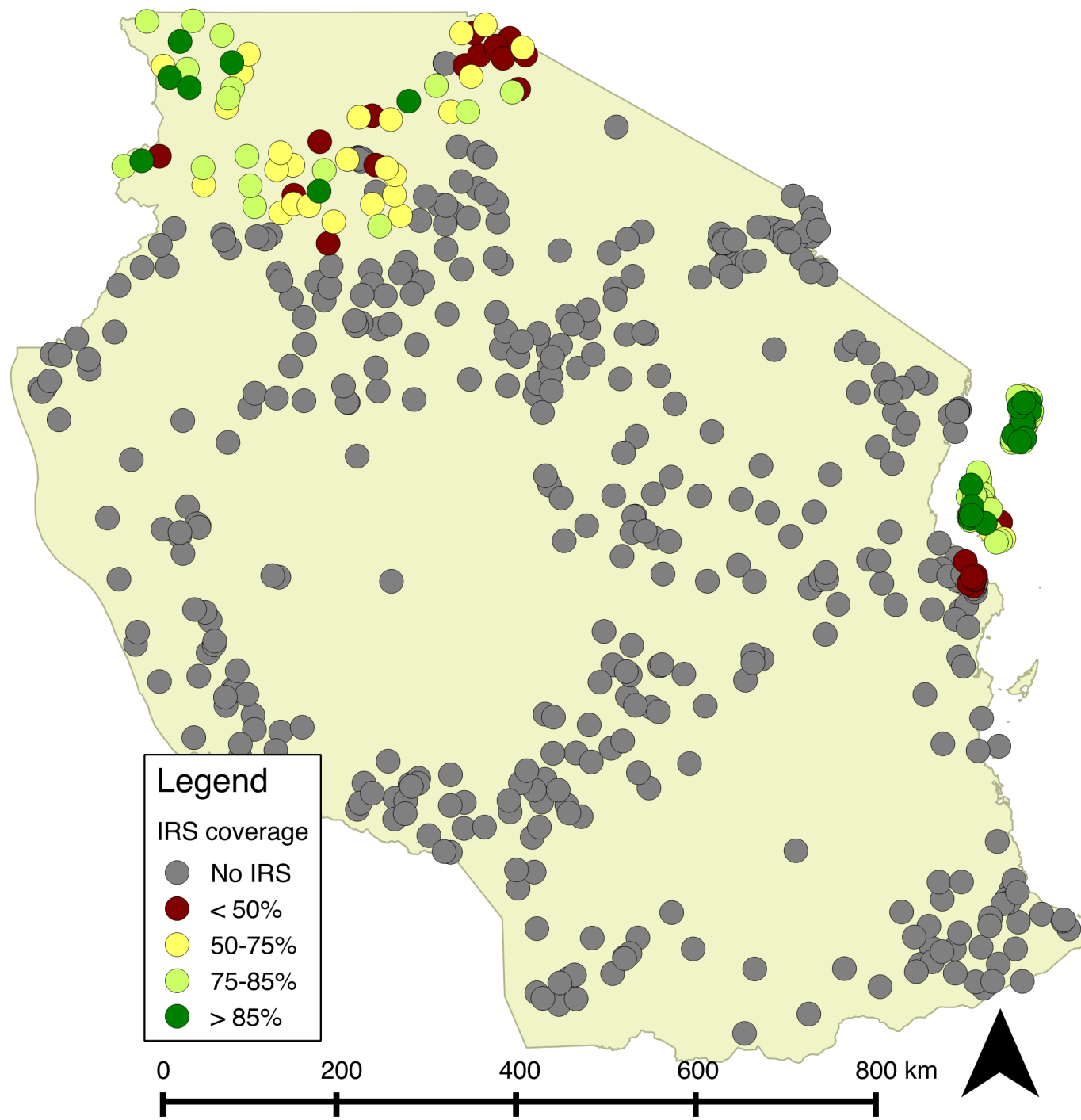

Supplement: Supplementary file 24 — Additional file 24. Map showing IRS coverage by community in Tanzania 2011. [file 12936_2017_1893_MOESM24_ESM.pdf]

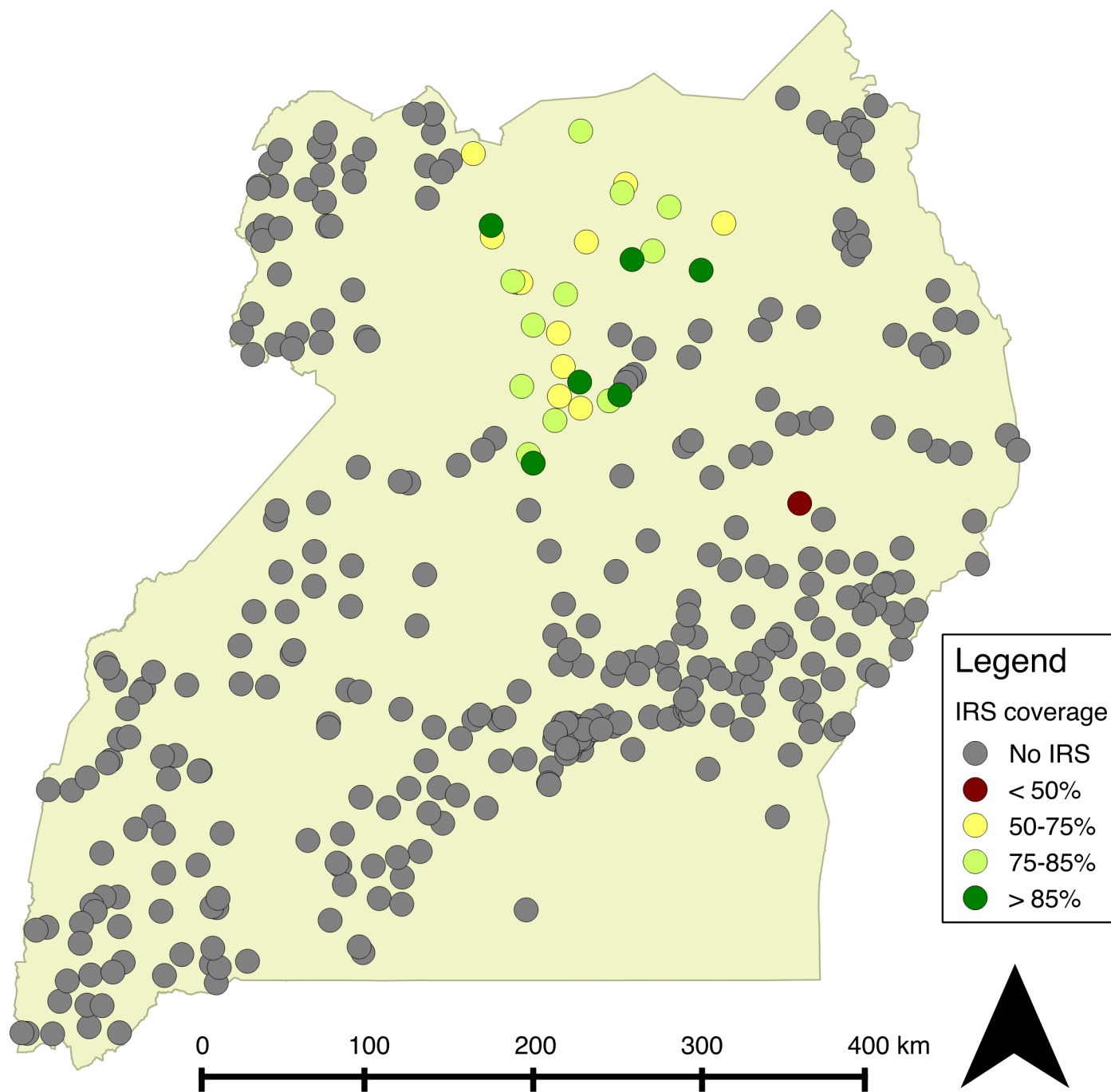

Supplement: Supplementary file 25 — Additional file 25. Map showing IRS coverage by community in Uganda 2011. [file 12936_2017_1893_MOESM25_ESM.pdf]

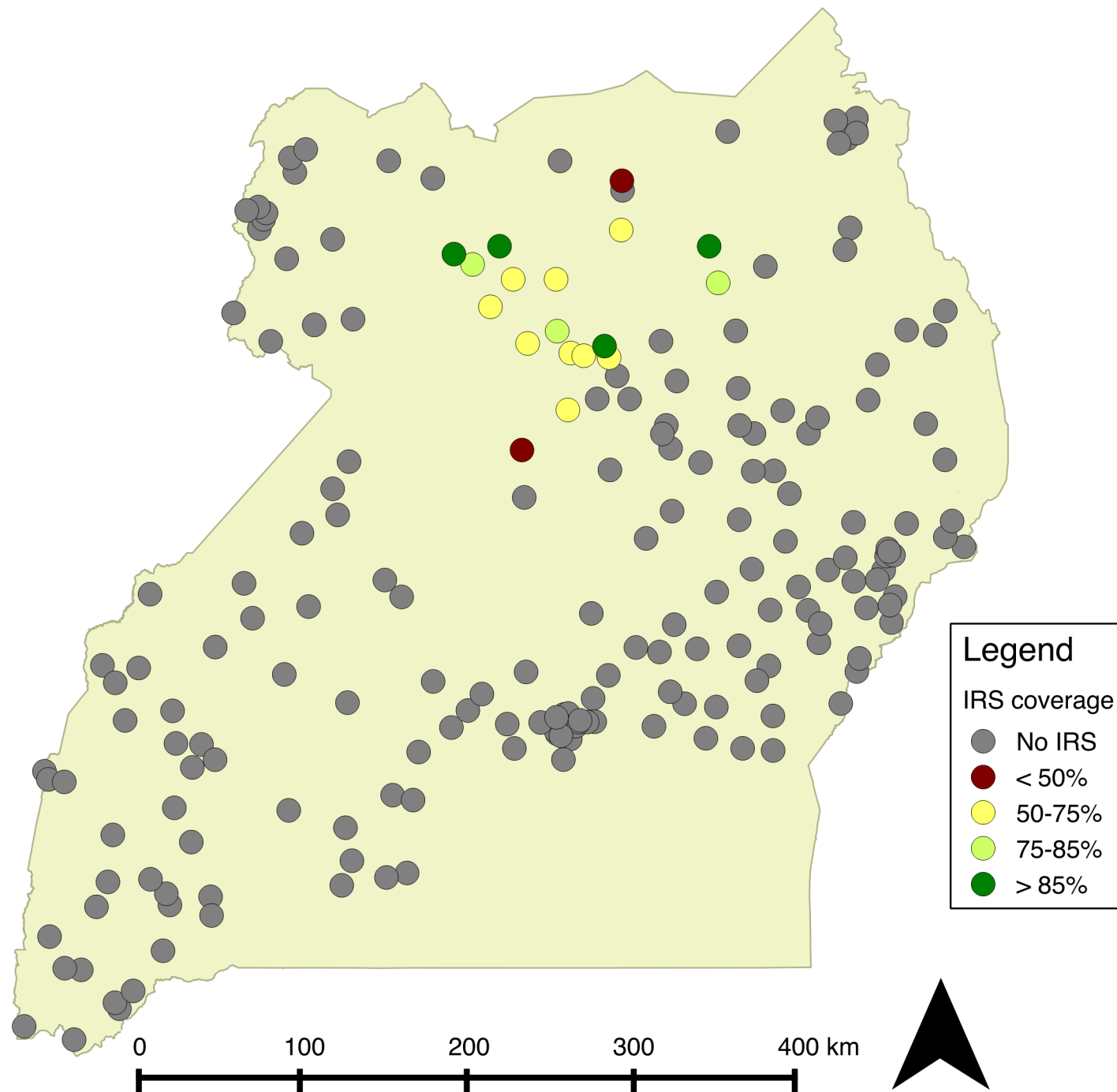

Supplement: Supplementary file 26 — Additional file 26. Map showing IRS coverage by community in Uganda 2014–2015. [file 12936_2017_1893_MOESM26_ESM.pdf]

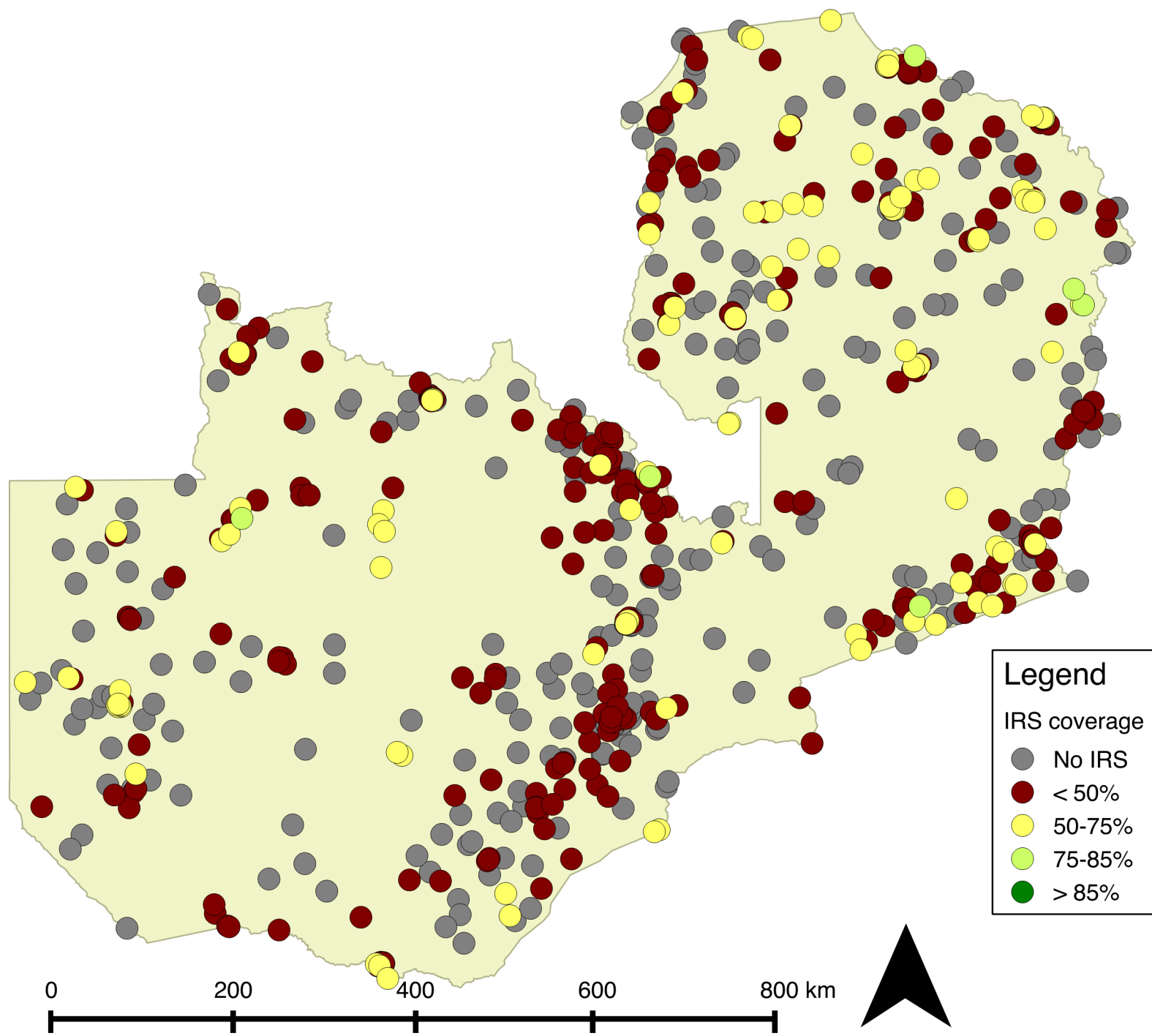

Supplement: Supplementary file 27 — Additional file 27. Map showing IRS coverage by community in Zambia 2013. [file 12936_2017_1893_MOESM27_ESM.pdf]

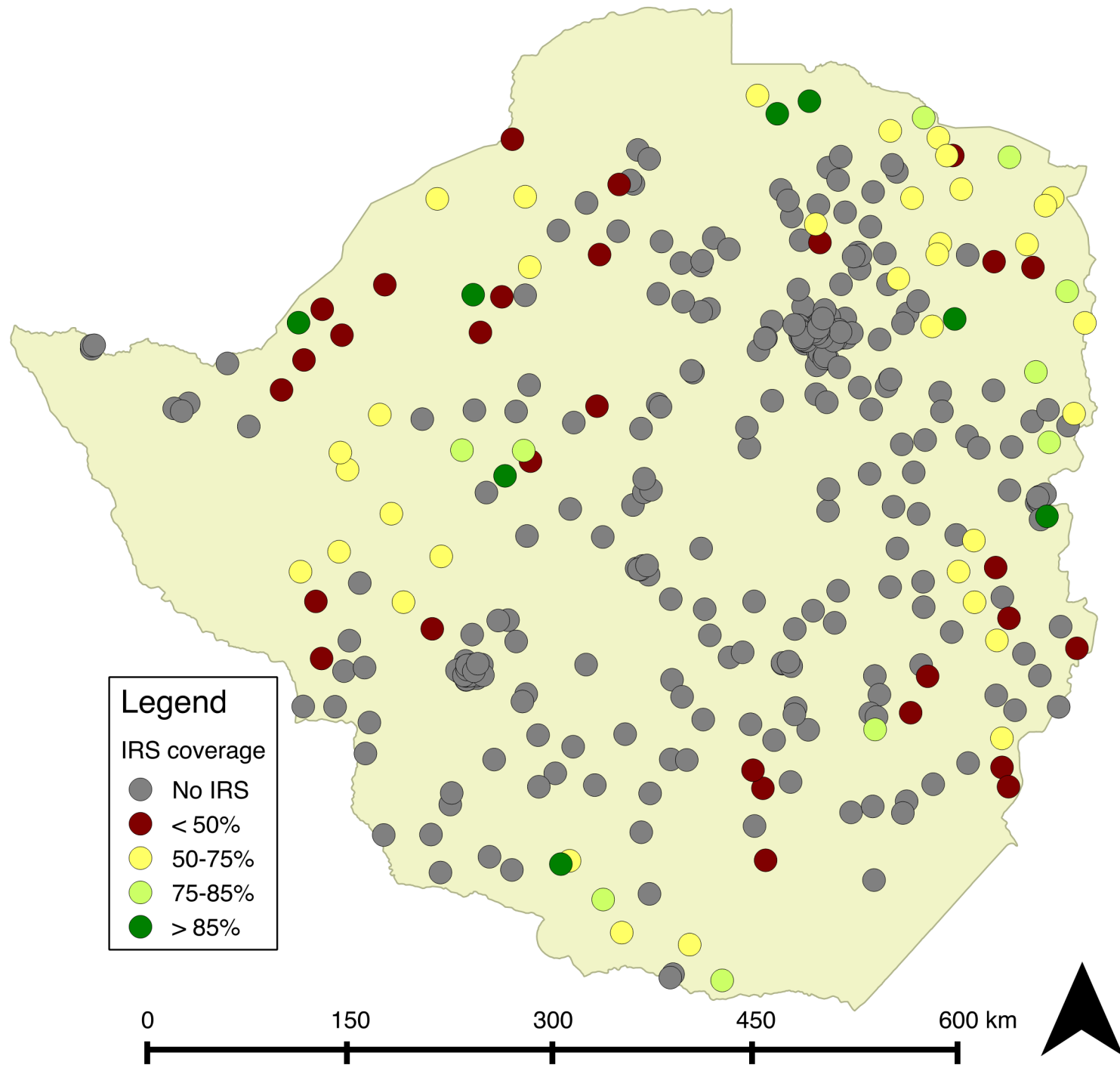

Supplement: Supplementary file 28 — Additional file 28. Map showing IRS coverage by community in Zimbabwe 2010–2011. [file 12936_2017_1893_MOESM28_ESM.pdf]
